# Outcomes of deprescribing for people with life-limiting conditions: A systematic review

**DOI:** 10.1177/02692163261416281

**Published:** 2026-02-22

**Authors:** Rajeev Shrestha, Emily Shaw, Liam Mullen, David Sinclair, Felicity Dewhurst, Adam Todd

**Affiliations:** 1 NIHR Newcastle Patient Safety Research Collaboration, Newcastle University, Newcastle upon Tyne, UK; 2 Northumbria Healthcare NHS Foundation Trust, Newcastle upon Tyne, UK; 3 Population Health Sciences Institute, Newcastle University, Newcastle upon Tyne, UK; 4 St Oswald’s Hospice, Newcastle upon Tyne, UK; 5 School of Pharmacy, Faculty of Medical Sciences, Newcastle University, Newcastle upon Tyne, UK

**Keywords:** deprescription, inappropriate medication, medication optimisation, older adults, palliative care

## Abstract

**Background:**

Polypharmacy and inappropriate medication are common amongst people with life-limiting conditions. Whilst deprescribing may help reduce these medication-related issues, supporting evidence in this population group is limited.

**Aim:**

To synthesise evidence on the outcomes of deprescribing in people with life-limiting conditions.

**Design:**

Systematic review.

**Data source:**

MEDLINE, Embase, Scopus, PsycINFO and CINAHL were searched. Original studies published between Jan 2000 and Dec 2024 in English were included.

**Result:**

A total of 17,457 hits were screened, of which 46 original studies met the inclusion criteria. Most eligible studies were pre-post interventional (*n* = 14) or cohort studies (*n* = 14), conducted primarily in nursing homes or long-term care facilities (*n* = 20) and hospitals (*n* = 16). The majority originated from North America (*n* = 20) and Australia (*n* = 7). A wide range of outcome variables were examined, with a primary focus on clinical outcomes. All studies assessing the impact on the number of medications used reported either a reduction in overall medication burden or inappropriate medications (*n* = 15), or no significant change (*n* = 3). Regarding mortality, most studies (10 studies) reported no impact, while 3 studies each reported increased and decreased mortality. For other outcomes, the majority of studies reported that deprescribing had no effect.

**Conclusion:**

This systematic review suggests that deprescribing offers some benefits, including reduced medication burden and costs in people with life-limiting conditions. While there is no strong evidence for harm, a small proportion of patients reported increased risks, so careful monitoring is essential. Further research should explore how deprescribing outcomes vary by disease condition and medication type.

**What is already know about the topic?**

- Polypharmacy and inappropriate medication use is common in people with life-limiting conditions, and is exacerbated by the addition of symptom management medication to a legacy of preventative treatments.
- While deprescribing can reduce the medication burden, it remains a complex and challenging intervention in people with life-limiting conditions.

**What this paper adds?**

- A broad range of deprescribing outcomes are reported across clinical-, medication- and system-related impacts.
- Existing studies are mostly from the US and Australia with little representation from low-and middle-income countries.
- Deprescribing is generally safe, with most studies demonstrating no negative effects. However, careful monitoring is recommended to mitigate potential risk in minority of cases.

**Implication for practice, theory, or policy**

- Careful monitoring is essential while implementing deprescribing interventions in individuals with life-limiting conditions.
- Further research is needed to strengthen the evidence base for deprescribing, particularly in relation to specific diseases and medication classes.

## Introduction

Life-limiting conditions are progressive, incurable illnesses that are expected to shorten a person’s life, such as cancer, organ failure, and neurodegenerative conditions.^1,2^ People with life-limiting conditions frequently experience polypharmacy and the use of inappropriate medications.^3–7^ Previous studies have reported polypharmacy and potentially inappropriate medication use in people with advanced cancer^8,9^ and dementia,^10–12^ as well as people receiving palliative^3,13,14^ or specifically, hospice care.^15,16^ In people with life limiting conditions, the presence of multiple long-term conditions and other age-related factors (e.g. frailty), can further increase the risk of polypharmacy and the use of potentially inappropriate medications.^5,17^ It has also been shown that polypharmacy and the use of inappropriate medication can adversely affect patients’ health outcomes, including adverse drug events, reduced medication adherence, hospitalisation, and increased risk of morbidity and mortality – although studies have not specifically focussed on people with life-limiting conditions.^18–21^

For people with life-limiting conditions, medication optimisation can be challenging for a number of reasons, including; the complex and dynamic medical needs of such patients, the potential psychological impact of changing medication – something which is relevant for both patients and caregivers, as well as the risk of symptom exacerbation upon reducing or stopping medication.^22–25^ In this context, it is important that medication use aligns with the evolving health status and goals of care of the patient.^26^

One potential solution to the challenge of polypharmacy and potential inappropriate medication use is deprescribing. Deprescribing aims to reduce medication burden, specifically ensuring discontinuation of inappropriate medications.^27–29^ It is a systematic process of identifying and discontinuing medications that are no longer beneficial, or where the risks outweigh the intended benefits. Previous studies have reported that deprescribing can improve survival or reduce mortality risk in specific contexts, such as among early older adults (aged 65–79 years)^30^ or older patients in end-of-life care.^27–31^ The impact of deprescribing on other health-related outcomes (e.g. falls or hospitalisation), or among vulnerable patient groups (e.g. people with frailty or dementia) remain limited.^27,29^

In the context of people with life-limiting conditions, evidence for deprescribing to improve patient outcomes is lacking.^27,31–33^ Previous systematic reviews, published by Shrestha *et al.* in 2020^33^ and 2021,^31^ reviewed deprescribing outcomes amongst older people with limited life expectancy and concluded there was evidence that deprescribing improved medication appropriateness; the review also concluded that evidence needs to be better established for other outcomes. Another systematic review, published in 2021, examined the effect of deprescribing interventions in older adults close to end-of-life, using the Criteria for Screening and Triaging to Appropriate aLternative care (CriSTAL) risk prediction tool,^32,34^ concluded it was difficult to ascertain if deprescribing improved patient outcomes. Since the publication of these systematic reviews, there has been an exponential increase in deprescribing research; for examples Hurley *et al.*,^35,36^ (2024), Etherton-Beer *et al.*^37^ (2023), Tapper *et al.*^38^ (2022), Niznik *et al.*^39,40^ (2020). As such, a comprehensive updated systematic review exploring evidence on deprescribing outcomes among older people with life-limiting conditions is warranted. This study aimed to address this gap and examine the evidence for outcomes of deprescribing for people with life limiting conditions.

## Methods

The protocol for this systematic review was developed and registered in the Prospective Register of Systematic Reviews (PROSPERO; CRD42024622342). This review is reported to the Preferred Reporting Items for Systematic Reviews and Meta-Analyses (PRISMA) guideline (Supplemental Table 1).

## Inclusion criteria

The population, intervention, comparison, outcome, study design (PICOS) framework was used to conceptualise the review inclusion criteria (Table 1). To be eligible for inclusion, studies had to be published in English, from January 2000 to December 2024. The date restrictions were implemented to ensure the inclusion of recent and relevant research that accurately reflects current (de)prescribing practices.

**Table 1.** The PICOS framework used for the systematic review.

PICOS	Description
Population	**People with life-limiting conditions:** People with advanced or end stage disease or conditions such as advanced cancer, organ failure (heart, lung, kidney, liver), neurodegenerative disease, multiple long-term conditions and frailty. Advanced or end stage diseases are indicated by the presence of any of the following indicators: ➢ Functional decline/deteriorating trajectory ➢ The presence of crisis events, such as frequent falls, hospital admissions ➢ Requiring assistance for instrumental daily activities ➢ Progressive weight loss ➢ Positive response to surprise question and other indicators provided by Gold Standard Framework and British Geriatric Society ➢ Those under hospice/palliative care (either specialist or general)
Intervention	**Deprescribing intervention:** A process of reducing or stopping of medication.
Comparison/control	Where applicable, studies with a comparator included usual care or non-deprescribing. However, studies without a direct comparator were also included if they had provided relevant insights into deprescribing outcomes.
Outcome	• Clinical outcomes: (e.g. falls, frailty, cognition, depression scores, quality of life). • Medication outcomes: (e.g. changes in prescribing, number of potentially inappropriate medications, medication adherence, drug interactions, adverse drug reactions). • System outcomes: (e.g. frequency of hospital or emergency visit, referral cases).
Study design	Both interventional studies (e.g. randomised controlled trials) and observational studies (e.g. cross sectional and cohort studies) included.

## Search strategy

Following the PICOS refinement, a search strategy was developed. Relevant keywords and controlled vocabulary with appropriate synonyms and Boolean logic were used. MEDLINE, Embase, Scopus, PsycINFO and CINAHL were systematically searched to identify relevant literature. The literature search was supplemented by forward citation searching of relevant studies. Detailed search strategies employed for each database are provided (Supplemental Table 2 to 6).

## Study selection and data extraction

Two reviewers (RS and LM) independently screened titles and abstracts for eligibility. Any disagreement during this stage was resolved through discussion between the two reviewers. No studies were excluded at this stage without mutual agreement. Following this, the same reviewers (RS and LM) independently assessed full text articles for eligibility. Any uncertainties that arose were discussed with the clinical members of the research team (AT, a pharmacist and FD, a palliative medicine doctor) who had final consensus.

A standardised data extraction form was developed to capture information on study characteristics, population characteristics, intervention descriptions including medication details (method of deprescribing, medications deprescribed, duration, follow-up, pattern of medication use) and outcomes (information on clinical, medication, and system-related outcomes). Corresponding authors of eligible papers were contacted for further clarification, if required. The data were extracted in full by one author (RS) and checked by a second author (ES). Any disagreement was discussed between RS and ES and, if agreement could not be reached AT had consensus as the senior author.

## Quality appraisal

Joanna Briggs Institute critical appraisal tools were used to assess the quality of included studies. This tool provides a comprehensive suite of tools tailored to different study designs aligning with established evidence synthesis methodologies, with recent revisions focussing on evaluating the risk of bias.^41–43^ RS assessed the risk of bias of the included studies which was then checked, in full, by ES. Any conflicts were resolved through discussion with the senior author (AT) who had consensus. No studies were excluded based on the results of the critical appraisal. All studies meeting the eligibility criteria were included to provide a comprehensive overview of the available evidence.

## Data synthesis

Due to the considerable heterogeneity in studies of interest (e.g. different study designs, patient populations, interventions and reported outcomes), a descriptive synthesis approach was undertaken to analyse the data, grouped by theme. Outcomes of deprescribing were organised into common groups under three broad categories: clinical-, medication- and system- related outcomes. Extracted data were summarised in tabular format to facilitate comparison across studies.

When a study reported multiple measures using a validated or stated instrument or scale for a single outcome domain, each measure was listed separately in the summary table. For example, if a study assessed cognitive function using (i) the Mini-Mental State Examination (MMSE) scale and (ii) the Cognitive Performance Scale (CPS) in across the intervention and control groups, all four measurements (i.e. two outcomes from each group) were reported as separate data points. Similarly, when a study reported outcomes separately for distinct patient subgroups over for total patients, each subgroup was treated as a separate data point in the synthesis. For example, in studies that presented deprescribing outcomes separately for patients with dementia and without dementia, the data from each group was extracted and reported as separate entries. The effects of deprescribing on each outcome, as reported by the included studies, were categorised into three groups:

- Positive effect: Studies reporting improvement or beneficial effects for patients with statistical analysis for significance were categorised as positive effects (e.g. reduced medication burden, improved clinical parameters, reduced adverse drug events).
- No effect: Studies showing no statistically significant changes were categorised as no effect.
- Negative effect: Studies reporting potential harm or worsening of outcomes for patients with statistical analysis for significance were categorised as negative effects (e.g. increased adverse drug events, symptom deterioration, increased hospitalisations).

For example, if a study reported a statistically significant lower MMSE for the deprescribing group compared to control group, it was categorised that deprescribing had a ‘Negative effect’ on cognitive function.

For findings reported without statistical analysis for significance, the categorisation into positive, negative, or no effect was based on the direction of the reported trend or the authors’ interpretation. For example, if a study reported a reduction in the number of patients with falls after deprescribing intervention without performing statistical analysis for significance, it was also categorised as a ‘Positive effect’ in the summary table.

To distinguish findings with and without statistical analysis for significance, an indication mark was given in the summary table. The term ‘significant’ in the result section refers to the outcome with statistical analysis for significance.

## Results

### Study selection

**Figure 1.**
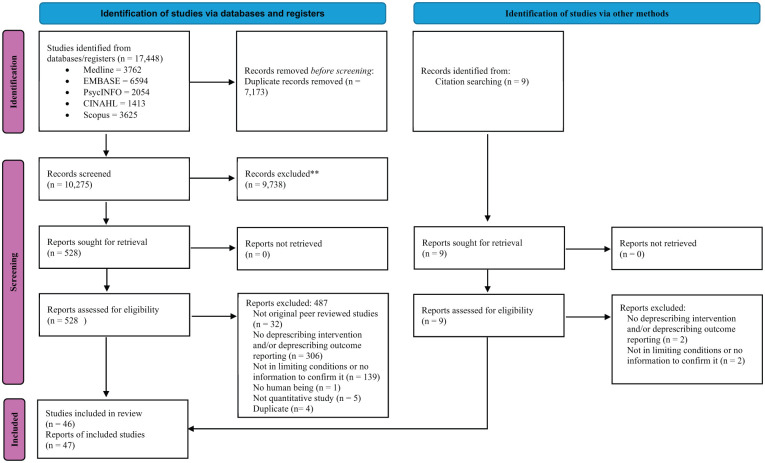
PRISMA flowchart of included studies.

Database and reference searching identified 17,457 records. After removal of duplicates, 10,284 records remained for title and abstract screening. Of these, 537 progressed to full-text review, resulting in 46 original studies meeting the inclusion criteria. The study selection process is detailed in Figure 1.

### Characteristics of included studies

**Figure 2.**
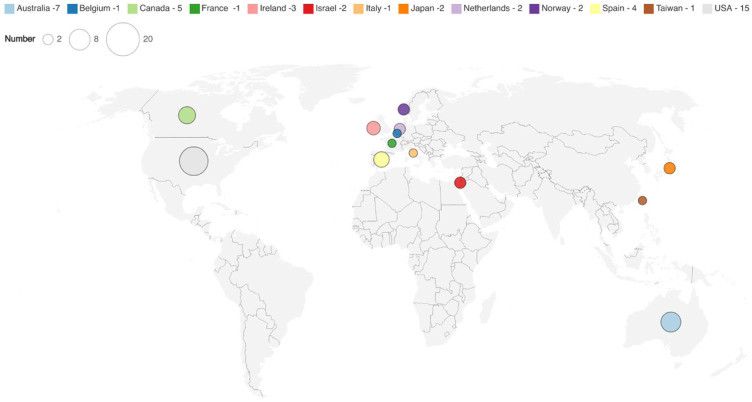
Map of geographical locations of included studies.

Among the included studies, the most common study designs were cohort (*n* = 14),^39,40,44–55^ pre-post interventional (*n* = 14)^35,56–68^ and randomised control studies (*n* = 11).^37,69–78^ The majority of the included studies were conducted in nursing homes/long-term care facilities (*n* = 20)^35–37,39,40,44,46,48,52,60,61,63,67,71,72,73,74–77,84^ hospitals (*n* = 16)^45,47,51,53–55,57,59,64,65,68,69,78,79,80,81^ and hospice/palliative care units (*n* = 5).^49,56,62,68,70^ Studies were also conducted in specialist care clinics (*n* = 2),^58,82^ a telemedicine palliative care clinic (*n* = 1),^66^ and home-based palliative care settings (*n* = 1).^50^ Most studies were conducted in the US (*n* = 13),^38,40,44,46–48,58,59,62,66,67,70,71^ and Australia (*n* = 7)^37,54,60,65,74,77,79^ (Table 2 and Figure 2).

**Table 2.** Characteristics of included studies.

Author, year, country	Study design, study setting and duration	Selection criteria	Details of participants				Details of Deprescribing intervention group				Comparator	Conflict of Interest and Funding
			Sample, age and gender	Primary disease condition	Polypharmacy and inappropriate medication	Deprescribing Methods/approach taken	Deprescribing rate/metrics	Deprescribing duration	Re-prescribing of deprescribed medicine	Any other intervention on medications		
Basri *et al.*,^56^ 2018, US	Retrospective review of pre-post intervention (single group), Hospice inpatients, Duration: Sep to Dec 2016	Patients admitted to hospice unit	Intervention = 32, mean age 73.8, female 3.1%	Cancer (59.4%), end-stage dementia (18.8%)	NR	Discontinuation of medicine not indicated by Clinical pharmacy specialist in collaboration with multidisciplinary hospice team	83 (18.3%) out of 453 intervention was on deprescribing	during hospice stay	NR	Medication adjustment (on dosage and frequency) and addition	Pre-post study	NR
Hurley *et al.*,^36^ 2024a, Ireland	Descriptive cost avoidance study, Nursing homes, Duration: August 2021–April 2023	Frail older (⩾65 years) nursing home resident meeting STOPPFrail criteria^a^	Intervention = 69 Total enrolled = 99	Dementia (64.6%), Hypertension (53.5%)	176 PIMs in 69 patients (average 2.6)	STOPPFrail tool used for identification by pharmacist and with GP’s and nursing team review PIM discontinued	176 PIM identified in 69 patients deprescribed (average 2.6)	6 months	NR	No	None	No COI, and funded study
Curtin *et al*.,^69^ 2020, Ireland	RCT, acute hospitals, Duration: Mar 2018 to Apr 2019	Hospitalised older adults (⩾75) severely frail, requiring advanced long-term care, positive to ‘surprise question’	Intervention = 65, mean age 84.49, female 64.61% Control = 65, mean age 85.68, female 58.46%	Dementia (75.4% in I, 73.8% in C), atrial fibrillation (41.5% in C, 36.9% in I)	Average number of medication = 11.52 in I and 10.89 in C ( >10 medications in 70.8% (I) and 60% (C) patients) PIM per patients = 2.40 in I and 2.41 in C	STOPPFrail guide deprescribing presented to attending physicians for approval	87.8% of recommendations (2.4 and 0.75 medications reduction per patient) were accepted.	Not clear	NR	NR	Usual pharmaceutical care without STOPPFrail-guided deprescribing	COI stated, and funded study
Niznik *et al.*,^44^ 2022, US	Retrospective cohort study, Nursing Homes, Duration: from 2009 to 2015	Residents with limited life expectancy (<6 months) or advanced dementia and potentially overtreated for diabetes (HbA1c ⩽ 7.5% and one or more diabetes medications)	Intervention = 554, ⩾85 age 19.3%, female 0% Control = 554, ⩾85 age 19.3%, female 0%	Diabetes with hypertension (95.8%) and hyperlipidaemia (71.3%)	NR	Clinician examined patients and de-intensified medications (either decreasing the dose or discontinuing a non-insulin agent and/or discontinuing a type of insulin with no addition of new agents or dose increases)	All in intervention group de-intensified	At least 7 days	No	NR	No de-intensification	No COI, and funded study
Niznik *et al.*,^39^ 2020a, US	Retrospective cohort study, Nursing home, Duration:2015 to 2016	Older (⩾65) nursing home residents with severe dementia	Intervention = 11,567 Control = 64,416	Dementia with mechanically altered diet (49.1% in I, 52% in C) and Heart failure (15.6% in I, 15.7% in C)	Average regular medicine = 5 in I, 5.2 in C	Discontinuation of acetylcholinesterase inhibitors	All in intervention group discontinued	At least 1 month	No	NR	Continued use	No COI, and funded study
Brunet *et al.*,^57^ 2014, Spain	Non-experimental pre-post analysis at acute geriatric unit of hospital. Duration: May 2011 to April 2012	Patient with advanced dementia admitted to geriatric unit	Intervention = 73, 86.1 mean age, 79.45% female	Dementia with trauma (35.61%) and infection (36.98%)	Mean number of medications at admission was 7.27 (Polypharmacy in 82.2%)	Multidisciplinary team reviewed and discontinued medication based on available evidence in literatures	All in intervention group deprescribed (2.45 per patients)	Not clear	NR	Medication related problem and symptom management	Pre-post design	No COI, and funding not reported
Hurley *et al.*,^35^ 2024b, Ireland	Prospective interventional study, Nursing homes, Duration: Aug 2021 to Apr 2023	Older adults (⩾65) nursing home residents meeting STOPPFrail criteria^a^	Intervention = 99, 86.24 mean age, 76.7% female	Dementia (64.6%), Hypertension (53.5%)	Average medications = 10.9 (59.6%, *n* = 59 on >10 medications)	Pharmacist identified and recommended medication for deprescribing using STOPPFrail to physicians for approval	At least one PIM was deprescribed per patients (Median number of STOPPFrail recommendations accepted (IQR) = 2.0 (1.0–3.0))	6 months (167 remained deprescribed)	9 deprescribed medicine restarted	NR	Pre-post design	No COI, and Not funded study
Niznik *et al.*,^40^ 2020b, US	Retrospective cohort study, Nursing home, Duration:2015 to 2016	Older (⩾65) nursing home residents with severe dementia	Participants = 37,106, ⩾80 aged 77.4%, female 75.5%	Dementia with mechanically altered diet (49.7% in I, 52.7% in C) and Heart failure (15.4% in I, 15.7% in C)	Average medication = 5.4 in I and 5.8 in C	Discontinuation of acetylcholinesterase inhibitors for a month	All in intervention group	At least a month	No	NR	Continued acetylcholinesterase inhibitors	No COI, and funded study
Kutner *et al.*,^70^ 2015, US	RCT, Palliative Care Research Cooperative Group member sites, Duration: Jun 2011 to May 2013.	Adults (⩾18 years) with advanced LLI and life expectancy 1–12 months, positive to surprise question	Intervention = 189, mean age 74.8, female 48.1% Control = 192, mean age 73.5, female 41.7%	Malignant tumour (44.4% in I, 53.1% in C)	Average non-statin medications = 11.6 for Intervention, 11.5 for Control	Discontinuation of statin directly	All in intervention group discontinued	12 months	NR	NR	Continued statin	No COI, and funded study
Tse *et al.*,^71^ 2008, US	RCT, Nursing Home, Duration:4 weeks	Patients with advanced Parkinsonism and dementia residing in nursing homes.	Intervention = 6, mean age 80.83, female 50% Control = 5, mean age 85, female 20%	Both parkinsonism and Dementia (100%)	NR	Dopaminergic medications were slowly tapered and then withdrawn	All in intervention group deprescribed	1 month	NR	NR	Continued medications	NR
M Chess-Williams *et al.*,^79^ 2024, Australia	Observational descriptive study, hospital, Duration: Oct 2020 to Mar 2021	Life-limiting patients referred to specialist palliative care community telehealth service	95 patients, mean age 75.18, 41% female	Metastatic cancer (69.5%), localised cancer (18.9%)	Average number of medications = 10.47, 88.4% patients in polypharmacy, 56.8% taking PIM (average 2.6)	Pharmacist identified and recommended medication for deprescribing using STOPP/Frail to attending doctor for approval	51.0% (25/49) of the deprescribing recommendations were accepted	For 6 months	NR	Medication related problem, symptom management and medication administration	None	No COI, and funded study
Whitman *et al.*,^58^ 2018, US	Pre-post single group intervention pilot study, Geriatric oncology clinic, Duration: August 2015 to April 2016	Elderly (⩾65 age) cancer patients with multimorbidity	Intervention = 26, mean age 81, female 46%	Cancer (100%)	Average number of medications = 12	Deprescribing using Beers and STOPP criteria and MAI	87 medications discontinued in 26 patients (3 per patients)	Not Reported	Two medications restarted due to clinical need	NR	Pre-post design	No COI, and funding not reported
Bergh *et al.*,^72^ 2012, Norway	RCT, Nursing home, Duration: Aug 2008 to Jun 2010	Nursing home resident, dementia with neuropsychiatric symptom	Intervention = 63, mean age 85.3, female 78% Control = 65, mean age 86.1, female 72%	Dementia (100%)	NR	Antidepressant was tapered off or replaced with placebo	All in intervention group	1 week	NR	Changes in Psychotropic medication other than antidepressant allowed if needed	Antidepressant continued	COI stated, and funded study
Saad *et al.*,^59^ 2012, US	Retrospective review of pre-post intervention study (single group), Tertiary hospital, Duration: Jan to Oct 2008	Elderly with multimorbidity and frailty requiring geriatric consultation	Intervention = 62, mean age 84.6, female 79%	Hypertension (68%), Dementia (53%)	Average number of medications = 7.7 (± 3.7)	Geriatrician reviewed and discontinued some medicines	24 medicines discontinued in 62 patients	Intervention during admission period	NR	Medication addition (116 added) and medication adjustment (12 adjusted)	Pre-post design	No COI and not funded study
Frankenthal *et al.*,^b^ 2014^84^ & 2017,^73^ Israel	RCT, chronic care geriatric facility, Duration: April 2012 to Sep 2013	Elderly (⩾65), chronic care resident with higher dependency	Intervention = 183, 49.7% ⩾ 85 age, female 70.5% Control = 176, 43.8% ⩾ 85 age, female 62.5%	Hypertension (76.3% in I, 67.8% in C), Dementia (51.9% in I, 56.8% in C)	Average number of medications = 8.8 in I and 8.2 in C	STOP/START criteria used for deprescribing recommendation by pharmacist	82.4% of STOPP and 92.6% of START recommendation accepted	NR	NR	NR	No deprescribing recommendation	No COI, and funded study
Potter *et al.*,^74^ 2016, Australia	RCT, RACFs, Duration: July 2011 to Dec 2013	Elderly (⩾65), frail, low level of function and residing in aged care facilities	Intervention = 47, mean age 84, 55% female Control = 48, mean age 84, 48% female	Hypertension (64% in I, 67% in C), Osteoarthritis (53% in I, 58% in Control)	Average regular medicine = 9.6 in intervention, 9.5 in Control	planned cessation of non-beneficial medicines following the deprescribing algorithm protocol	89% (42/47) participants had at least one medicine deprescribed	Not clear	NR	NR	No deprescribing	COI stated, and funded study
Etherton-Beer *et al.*,^37^ 2023, Australia	RCT, RACFs, Duration, Mar 2014–Feb 2019	Elderly (⩾65) frail low level of function and resident of aged care facilities	Blind intervention = 102, mean age 85.8, 76% female Open intervention = 101, mean age 84.8, 75% female Blind control = 100, mean age mean age 85, 77% female	NR	Average regular medicine = 10.3 + 4.5 (10.1 blind intervention, 10.7 open intervention, 10.1 blind control.)	Structured, clinically supervised withdrawal of medicines using a deprescribing algorithm	2.7 and 2.3 medication deprescribed per participants in blind and open intervention groups	12 months	No	NR	Continued medication	No COI, and funded study
Poudel *et al.*,^60^ 2015, Australia	Prospective observational cohort (pre-post single group), RACF, Duration: Jan 2013 to Aug 2014	Aged care resident with dementia and MLTC referred for geriatric consultation	153, mean age 83, female 64.2%	Dementia (67.3%), depression (46.4%)	Average regular medicine = 9.6, (45.8% on 5–9 and 45.1% on >10 medicines), 58.2% received at least one high risk medicine	Geriatricians and Nurses provided comprehensive geriatric assessment with identifying high-risk medication following Beers, McLeod, Laroche, PRISCUS and Norwegian General practice criteria	9.8% medicines stopped and 2.5% dose reduced	NR	NR	Medication added (6.9%, *n* = 102)	Pre-post design	No COI, and funding not reported
Brunetti *et al.*,^45^ 2024, Italy	Retrospective cohort, Tertiary hospital, Duration: Jan 2014 to July 2018	Inpatient elderly (⩾75) with atrial fibrillation, MLTCs and higher dependency	Intervention = 341, median age 86, female 57.5% Control = 1237, median age 85, female 55.9%	Atrial fibrillation (100%)	NR	No prescribing of oral anticoagulant therapy during discharge	All in intervention group deprescribed	NR	No	NR	Prescribing of oral anticoagulant therapy	No COI, and funded study
Nakagaito *et al.*,^51^ 2024, Japan	Prospective cohort study, Hospital, Duration: Feb 2016 to July 2022	Hospitalised for heart failure,	Intervention = 51, mean age 76, female 47% Control = 161, mean age 72, female 29%	Heart failure patients with diabetes mellitus (80% in I, 66% in C)	NR	Discontinuation of sodium-glucose cotransporter 2 inhibitors (SGLT2i) after hospitalisation	All in intervention group deprescribed	NR, 17 median days	NR	Adjustment of other heart failure medication	Continuation of (SGLT2i) after hospitalisation	No COI, and funding study
Caravaca *et al.*,^55^ 2018, Spain	Retrospective cohort study, Hospital outpatient, Duration: Jan 2013 to Dec 2015	Advanced chronic kidney disease (stage 4 or 5)	Intervention = 67, mean age 62, female 43% Control = 67, mean age 63, female 40%	Advanced CKD with Diabetes (49%)	NR	Structured discontinuation of vitamin D analogues (calcitriol, paricalcitol or 22-oxacalcitriol)	All in intervention group deprescribed	NR	No	NR	Pre-post design	No COI, and funding not reported
Tapper *et al.*,^38^ 2022, US	Emulated clinical trial, Medicare enrolees, Duration: 2008 to 2019	Elderly (median 68, ⩾65), compensated cirrhosis,	Intervention = 728, mean age 68.4, 57% female	Cirrhosis (100%)	NR	Complete deprescribing benzodiazepines for 90 days following cirrhosis diagnosis	All in intervention group deprescribed	3 months	No	NR	Continue use of traditional BZD and Zolpidem	COI stated, and funded study
Ruths *et al.*,^75^ 2004, Norway	RCT, Nursing home, Duration: Sep to Oct 2002	Elderly (⩾ 65) nursing home resident with dementia	Intervention = 15, Control = 15, Overall, mean age 83.4, female 80%	Dementia (100%)	Patients were receiving the median (range) daily doses of risperidone 0.5 (0.5–2.0) mg, olanzapine 5.0 (2.5–5.0) mg, and haloperidol 0.75 (0.5–1.0) mg.	Antipsychotic was disrupted from the end of baseline period	All in intervention group deprescribed	1 month	Restarted in one patient after 9 days	NR	Continued antipsychotics	NR
Bogaerts *et al.*,^76^ 2024, Netherland	RCT, Longterm care residents, Duration: Nov 2018 to May 2021	Long-term care resident with moderate-to-severe dementia and low level of function	Intervention = 101, median age 85.3, female 76.2% Control = 104, median age 86.6, female 82.7%	Dementia with Hypertension (100%)	Median no of drugs = 10	Stepwise, semi-protocolised discontinuation of antihypertensive medication	All in intervention group deprescribed	6 weeks	5.8% restarted medicine at 32 weeks follow up	NR	Continued use	COI stated, and funded study
Gerardi *et al.*,^82^ 2022, Canada	Quasi-experimental study, Haemodialysis clinic, Duration: Nov 2018 to Sep 2019	Patients under haemodialysis	Intervention = 66, mean age 72.4, female 27.3%	Dialysis patient with Hypertension (87.8%) and Diabetes mellitus (77.3%)	Average regular medication = 12.2,	Evidence based algorithm developed and used for deprescribing intervention	59.3% (*n* = 35) inappropriate medicine deprescribed (⩾1 per patients)	Variable (4–6 weeks)1 month	Deprescribing reintroduced in 6 patients	NR	Pre-post design	No COI, and funding not reported
Garfinkel *et al.*,^83^ 2007, Israel	Quasi-experimental, Geriatric medical Centre, Duration: 12 months/early 2004	Elderly inpatient with dementia and multiple comorbidities receiving palliative care	Intervention = 119, mean age 81.2, female 73% Control = 71, mean age 82, female 62%	Dementia (94% in I, 93% in C), Hypertension (46% in I, 41% in C)	Average number of medications = 7.09	Deprescribing algorithm followed for deprescribing	All in intervention group deprescribed (2.8 medicines per patients)	1 year	21 patients re-administered	NR	No discontinuation	NR
Malik *et al.*,^47^ 2019, US	Observational cohort, hospital, Duration March 2003 to December 2004	Hospitalised heart failure patients	Intervention = 698, mean age 76, female 40% Control = 698, mean age 76, 41% female,	Heart failure with Hypertension (64% in I, 66% in C) and Atrial fibrillation (45% in I, 45% in C)	Loop diuretics (73%) and beta-blockers (65%) mostly used	Digoxin discontinued before hospital discharge	All in intervention group deprescribed	NR	NR	NR	Continued use	No COI, and funded study
Daiello *et al.*,^46^ 2009, US	Retrospective cohort, Nursing home, Duration: Jan 2004 to Dec 2005	Nursing home resident aged ⩾ 60 with dementia	Intervention = 62, mean age 85.3, female 80.7% Control = 116, mean age 85.9, female 80.2%	Dementia with cardiovascular disease (87.1% in I, 85.3% in C)	Number of daily medications = 11 in I and 10.5 in C	Discontinued cholinesterase inhibiting agents	All in intervention group discontinued	2 months	No	NR	Continued use	NR
Wauters *et al.*,^61^ 2021, Belgium	Single pre-post interventional pilot study, Nursing home, Duration: Oct 2019 to Mar 2020	Nursing home dementia residents with higher dependency	100 participants, mean age 87.7, female 78%	Dementia (50.7%)	Average regular medicine = 5.6	Medication deprescribing recommended utilising Beers’, STOP/START, EU-PIM list and MARANTE scoring system	Changes recommended by pharmacists in 32 patients (32%) was approved	Not Clear	Discontinuation interrupted in 2 patients	Medicine adjustment (9 times)	Pre-post design	No COI, and funded study
Okafor *et al.*,^77^ 2024, Australia	RCT, RACFs. Duration: Mar 2014 to Feb 2019	Elderly (⩾65) frail low level of function and resident of aged care facilities	Blind intervention = 102, mean age 85.8, 76% female Open intervention = 101, mean age 84.8, 75% female Blind control = 100, mean age mean age 85, 77% female	NR	Average number of medications = 10.3 + 4.5 (10.1 in IG-blind, 10.7 in IG-open, 10.1 in CG)	Structured, clinically supervised withdrawal of medicines using a deprescribing algorithm	2.7 and 2.3 medication deprescribed per participants in blind and open intervention groups	12 months	No	NR	Continued medication	No COI, and funded study
Suhrie *et al.*,^62^ 2009, US	Retrospective review of pre-post interventional study (single group), Geriatric Palliative care unit, Duration: Aug 2005 to July 2007	Palliative unit patients	Participants = 89, mean age 79.7, female 2.2%	Dementia (39.3%), Cancer (16.9%)	Average number of medications = 9.7	Multidisciplinary team review with utilising MAI to identify unnecessary medicines	Average of 1.1 medicine per patients deprescribed	NR	NR	NR	Pre-post design	NR
Yeh *et al.*,^52^ 2013, Taiwan	Prospective cohort study, Veteran Home, Duration: 12 weeks	Dementia residents of Veteran Home	Intervention = 40, mean age 83, female 0% Control = 27, mean age 84, female 0%	Dementia (100%)	Average number of medications = 4.6 in I and 5.4 in C	Physicians of Intervention groups educated to tapering off or switching anticholinergic medications	NR	NR	NR	Medication prescribing as per need	No education to physician for deprescribing anticholinergics	No COI, and not funded study
Czikk *et al.*,^53^ 2022, Canada	Prospective cohort, Tertiary hospital, Duration: Jun to Oct 2021	End-stage kidney disease under haemodialysis	Intervention = 29, mean age > 65	End-stage kidney disease (100%)	NR	Proton pump inhibitors were gradually discontinued	NR	2 weeks	14 patients restarted	NR	Pre-post design	COI stated, and funded study
Hayes *et al.*,^48^ 2023, US	Retrospective cohort, Nursing home. Duration: Jan 2013 to Dec 2017	Long stayed (>100 days) nursing home resident (⩾65 aged) with nonvalvular atrial fibrillation and MLTCs	Intervention = 10,514, mean age 82.3, female 66% Control = 11,364, mean age 81.7, female 66.8%	Nonvalvular atrial fibrillation (100%)	6–10 medications in 29.3% in I, 28.1% in C 11 or more medications in 45.1% in I, 44.6% in C	Reduced dose therapy of direct oral anticoagulant	All in intervention group received reduced dose	NR	No	NR	Standard dose therapy	COI stated, and funded study
Whitty *et al.*,^80^ 2018, Canada	Quasi-experimental pilot study, Hospital, Duration: Aug to Dec 2015	Seriously ill or frail elderly patients at risk of 6-month mortality or ICU admission, or followed by the palliative care service	Intervention = 53, mean age 79.6, female 43% Control = 51, mean age 79.2, female 63%	Respiratory (23%) and cancer (19%) disease	Average medication use = 13.3 in I, 10.9 in C	Guideline-based algorithm used to deprescribe by interprofessional medication rationalisation (MERA) team	3.1 medication stopped per patients in 51 patients	During hospitalisation	25% (40) discontinued medication restarted	Addition of medication and dose changes	No structured deprescribing by MERA	COI stated, and funded study
Riveras *et al.*,^49^ 2024, Netherland	Retrospective cohort observational study, medical centre, Duration: 2021	Palliative cancer patients (>18 age), <3 months life expectancy using antithrombotic	Intervention = 80 Control = 31 Common median age = 70	Cancer (100%)	NR	Algorithm based approach to deprescribe antithrombotic	All in intervention group	During hospitalisation	No	NR	No structured review and discontinuation	No COI, and funded study
Chin-Yee *et al.*,^50^ 2022, Canada	Retrospective cohort study, home palliative care, Duration: 2010 to 2018	Elderly (> 65 age), MLTC receiving home palliative care and taking anticoagulant	Intervention = 2123, mean age 81.2, female 49.7% Control = 6564, mean age 81.2, female 53.5%	Hypertension (81.1% in I, 82.4% in C) and Cancer (79% in I, 77.5% in C)	NR	Discontinuation or gap in anticoagulant re-prescribing	All in intervention group	At least 7 days	No	NR	Continuation without gap	COI stated, and funded study
Bravo-Jose *et al.*,^63^ 2019, Spain	Prospective pre-post interventional study, Nursing Home. Duration: 1 year	Elderly nursing home residents with dementia	Intervention = 35, mean age 82.31 female 60%	Dementia (100%)	NR	Algorithm protocol-based approach to deprescribe antipsychotics	All in intervention group deprescribed (discontinuation in 28 and dose reduction in 7)	6 months	2 patients restarted due to worsening symptoms	NR	Pre-post design	No COI, and not funded study
Ferro-Uriguen *et al.*,^78^ 2023, Spain	RCT, Hospital, Duration: Feb 2018 to Feb 2020	Elderly (⩾65 years) hospitalised advanced chronic disease at end of life	Total participants = 81, mean age 87.3, female 58%	Dementia-like trajectory (55) and end stage organ failure trajectory (26)	Average number of medications = 7.6 in T1 and 9.7 in T2	Multidisciplinary team review with utilising STOPP Frail/Beers Criteria	NR	NR	NR	Medication review – dose adjustment, duplication checking, correct and practical directions, DDIs check	Usual care without multidisciplinary team review	No COI, and Not funded study
Choukroun *et al.*,^64^ 2021, France	Prospective observational (pre-post intervention) study, Hospital, Duration: May 2016 to Mar 2017	Cancer outpatients (>75 aged) referred for geriatric assessment	Intervention = 51, mean age 83, female 57%	Cancer (100%)	Median number of medication use = 10, polypharmacy in 80.4%	Multidisciplinary team reviewed and recommended for deprescribing to patient’s GP using STOPP/START criteria and Laroche list	36% of recommendations were for deprescribing	NR	NR	Medication addition, correction in dosing	Pre-post design	No COI, and funding not reported
Kearney *et al.*,^65^ 2023, Australia	Prospective pre-post interventional study, Hospital, Duration: 1 year	Advanced cirrhosis meeting palliative care criteria	Intervention = 30, mean age 64, female 43% Control = 30, mean age 63, female 13%	Advanced cirrhosis (100%)	Median no of medication = 13.5 (5–9 medication in 25, >10 medications in 20) in I	Multidisciplinary team reviewed and deprescribed	Deprescribing in 22 patients of IG	NR	NR	Correction on dosing error, medication addition	Usual care	No COI, and Not funded study
Ruderman *et al.*,^54^ 2018, Australia	Prospective observational cohort study, Hospital, Duration: Aug 2015 to Mar 2016	Dialysis patients with secondary hyperparathyroidism	Intervention = 51, mean age 69.6, female 45% Control = 51, mean age 68.6, female 45%	Kidney disease (100%)	NR	Cinacalcet withdrawal from intervention group	All of intervention group	12 months	Restarted patients (5) were excluded	None	No withdrawal of cinacalcet	COI stated, and funded study
Suzuki *et al.*,^81^ 2023, Japan	Multicentre prospective observation study, Hospital, Duration: Sep 2018	Hospitalised MLTC patients	Intervention = 347, 89.3% in their ⩾60	Cancer (85.6%)	Average regular medication = 8.2	Pharmacist reduces or discontinued inappropriate medications	Average number of discontinued drugs and dosage reduced are 1.7 and 0.6	NR	NR	None	None	No COI, and funding not reported
Shirley *et al.*,^66^ 2021, US	Prospective pre-post interventional study, palliative care clinic, Duration: Dec 2019 to May 2020	Veterans receiving palliative care	Intervention = 25, mean age 81, female 0%	Cancer (36%) and Dementia (28%)	Average medication number = 15.5	Comprehensive medication review and recommended for deprescribing using VA VIONE tool to consulting provider and/or primary treatment team	57% (72/126) recommendation accepted	NR	NR	Addressed medication-related concerns, educated medication compliance, medication education and medication adjustment	None	COI stated, and funding not reported
Pruskowski *et al.*,^67^ 2017, US	Prospective pre-post intervention study, Nursing home, Duration: Oct 2015–April 2016	Dementia with MLTC residing in nursing home	Intervention = 47, mean age 87.5, 88% female	Dementia (72%)	Average medication number = 9.63	Comprehensive medication review and recommended for deprescribing to	26% (10 out of 39) recommendation accepted	4 months	None	NR	Pre-post design	No COI and not funded study
McIntyre *et al.*,^68^ 2017, Canada	Prospective pre-post interventional study, tertiary-care haemodialysis unit, Duration: May 2014 to Mar 2015	Haemodialysis patient	Intervention = 35, mean age 65, female 60%	Kidney disease (100%)	Average medication number = 13.4	Algorithm based deprescribing recommendation	31 medicines deprescribed in 27 patients	6 months	5 medications restarted	NR	Pre-post design	No financial interest stated, and funding not reported

I: intervention: C: control; NR: not reported; NR: not reported; RCT: randomised controlled trial; LLI: life limiting illness; MLTC: multiple long term conditions; COI: conflict of interest; DDIs: drug-drug interactions; GP: general practitioners; IG: intervention Group; IG-Blind: blind intervention group; IG-Open: open intervention group; CG: control/comparator group; RACF: residential aged care facility; T1: dementia-like trajectory; T2: end stage organ failure trajectory; VA VIONE tool stands for vital, important, optional, not indicated/treatment complete, every medication has a diagnosis/indication.

a STOPPFrail Criteria: End stage irreversible pathology, Poor 1-year survival prognosis, Severe functional and/or cognitive impairment, Symptom control is the priority as opposed to prevention of disease progression.

b It has two publications of one study, both explored for relevant information.

**Figure 3.**
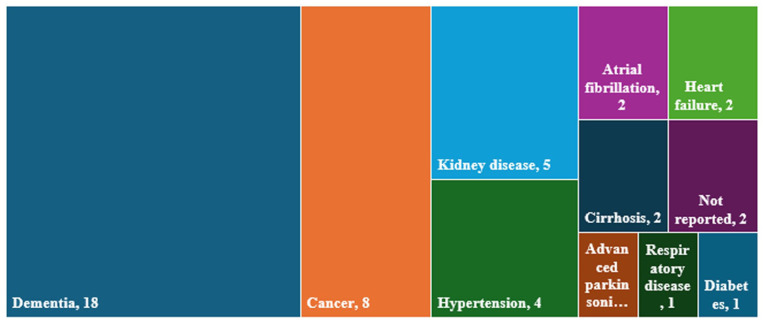
Primary disease conditions of included studies.

In terms of the population from the included studies, the majority were older adults who reported multiple disease conditions, taking a mean of 4.6^52^ to 13.3^80^ medications. The most reported disease conditions were dementia (18 studies)^35,36,39,40,46,52,57,60–63,67,69,72,75,76,78,83^ and cancer (8 studies)^49,56,58,64,66,70,79,81^ (Table 2 and Figure 3).

### Deprescribing characteristics

The deprescribing approaches undertaken among the included studies were heterogeneous. Most studies directly deprescribed targeted medicines^38–40,44–55,63,70–72,75,76^ and utilised a validated tool or criteria-based approach to identify inappropriate medication.^35,58,60–62,66,69,73,78,79^ Some studies used an algorithm-based approach, meaning they followed a structured, step-by-step decision-making process or flowchart, to identify medication for deprescribing,^37,68,74,77,80,82,83^ while other studies employed medication reviews without utilising any tools^56,57,59,65,67,81^ (Table 2).

Commonly reported deprescribed medications included acid suppressants, anti-thrombotics, oral hypoglycaemics, anti-hypertensives, lipid lowering agents, analgesics, psychotropics, dementia medications and supplements. Other reported deprescribed medications included anticholinergics, diuretics and antidepressants. A detailed description of the reported deprescribed medications is included as a Supplemental file (Supplemental Table 7).

### Deprescribing outcomes

Deprescribing outcomes were organised into three categories: clinical-, medication- and system-related outcomes (Table 3). The detailed outcomes reported by each study is available as a Supplemental file (Supplemental Tables 8–10).

**Table 3.** Summary of clinical, medication, and system-related outcomes reported by included studies.

Outcome		Positive effects	No effect	Negative effect
		‘*’ studies indicate finding without statistical analysis for significance and ‘*Italic’* font studies refers to RCTs		
Clinical-related Outcome	Cognitive and neuropsychiatric outcomes (9 studies, 26 data points)			
	Cognitive Functioning	-	**7 Studies (8 data points)** Daiello *et al.*,^46^ 2009 (Cognitive performance scale); *Bergh et al.*,^72^ *2012* (Clinical dementia rating and Severe impairment battery); *Etherton-Beer et al.*,^37^ *2023* (IG-Open MMSE); *Potter et al.*,^74^ *2016* (MMSE) ; Yeh *et al.*,^52^ 2013 (MMSE); *Bogaerts et al.*,^76^ *2024* (MDS-CPS); *Tse et al.*,^71^ *2008* (MMSE)	**1 Study (1 data points)** *Etherton-Beer et al.*,^37^ *2023* (IG-Blind MMSE)
	Frequency and Severity of Neuropsychiatric symptoms	-	**5 Studies (8 data points)** *Ruths et al.*,^75^ *2004* (NPI-Q); *Bergh et al.*,^72^ *2012* (10 item NPI); *Etherton-Beer et al.*,^37^ *2023* (IG-Open and IG-Blind −10 item NPI and IG-Open, and IG-Blind −12 item NPI); *Potter et al*.,^74^ *2016* (NPI-NH); *Bravo-Jose et al.*,^63^ *2019* (NPI-NH)	**1 Study (2 data points)** *Bogaerts et al.*,^76^ *2024* (NPI-NH and Short-CAM)
	Distress of Neuropsychiatric symptoms	-	**1 Study (4 data points)** *Etherton-Beer et al.*,^37^ *2023* (IG-Open and IG-Blind-10 item NPI Distress and IG-Open and IG-Blind-12 item NPI Distress)	**1 Study (1 data point)** *Bogaerts et al.*,^76^ *2024* (NPI-NH Caregiver distress)
	Behavioural Dysfunction with cognitive impairment	-	**1 Study (1 data point)** *Tse et al.*,^71^ *2008* (Nursing Assistant Behavioural Detection Form)	-
	Apathy level	-	**1 Study (1 data point)** *Bogaerts et al.*,^76^ *2024* **(**AES-10)	**-**
	Functional and physical health outcome (14 studies, 33 data points)			
	Falls	**2 Studies (2 data points)** Tapper *et al.*,^38^ 2022 (IG-Zolpidem),; Hurley et al.,^35^ 2024b*	**5 Studies (6 data points)** Tapper *et al.*,^38^ 2022 (IG-Benzodiazepam); *Curtin et.al.*,^69^ *2020; Frankenthal et al.*,^73^ *2017; Potter et al.*,^74^ *2016; Etherton-Beer et al.*,^37^ *2023* (IG-Open* and IG-Blind*)	**1 Study (1 data point)** *Bogaerts et al.*,^76^ *2024*
	Fractures	**2 Studies (3 data points)** Tapper *et al.*,^38^ 2022 (IG-Zolpidem); *Etherton-Beer et al.*,^37^ *2023* (IG-Blind* and IG-Open*)	**4 Studies (4 data points)** Tapper *et al.*,^38^ 2022 (IG-Benzodiazepam); *Curtin et.al.*,^69^ *2020; Potter et al.*,^74^ *2016*; Ruderman et al.,^54^ *2018*	-
	Fall or Fracture	**1 Study (1 data point)** Niznik *et al.*,^40^ 2020b	-	-
	Functional Independence in daily activities	-	**8 Studies (10 data points)** Daiello *et al.*,^46^ 2009 (ADL hierarchy scale); *Bergh et al.*,^72^ *2012* (Lawton and Brody’s physical self-maintenance scale); *Kutner et al.*,^70^ *2015* (Australia-Modified Karnofsky Performance Status score); *Frankenthal et al.*,^84^ *2014* (Functional Independence Measure score); *Etherton-Beer et al.*,^37^ *2023* (IG-Blind and IG-Open MBI); *Potter et al.*,^74^ *2016* (MBI); Yeh *et al.*,^52^ 2013 (Barthel Index); *Bogaerts et al.*,^76^ *2024* (CDS and Katz-15)	**1 Study (1 data point)** Daiello *et al.*,^46^ 2009 (time spend in leisure-related activities)
	Progression of Parkinson’s Disease	-	**2 Studies (2 data points)** *Tse et al.*,^71^ *2008* (UPDRS); *Bergh et al.*,^72^ *2012* (UPDRS)	**-**
	Motor and behavioural deterioration	-	**1 Study (1 data point)** *Tse et al.*,^71^ *2008* (Motor and Behavioural deterioration)	**-**
	Frailty	-	**1 Study (2 data points)** *Etherton-Beer et al.*,^37^ *2023* (IG-Open and IG-Blind Frailty index)	**-**
	**Behavioural and mood outcomes (5 studies, 12 data points)**			
	Behavioural Outcome	-	**1 Study (1 data point)** Niznik *et al.*,^39^ 2020a (aggressive behaviour scale)	**1 Study (4 data points)** Daiello *et al.*,^46^ 2009 (Aggressive behaviour scale, socially inappropriate or disruptive behaviour, Repetitive questioning, and Repetitive health complaints)
	Depressive symptoms	-	**1 Study (1 data point)** Daiello *et al.*,^46^ 2009 (Depression rating scale)	**1 Study (1 data point)** *Bergh et al.*,^72^ *2012* (Cornell scale of depression)
	Sleep quality	-	**2 Studies (4 data points)** Ruths *et al.*,^75^ 2004 (Total 24-h activity score, Day activity score and Night activity score); Potter *et al.*,^74^ 2016 (PSQI)	**1 Study (1 data point)** *Ruths et al.*,^75^ *2004* (sleep efficiency, %)
	Mortality and survival outcome (16 studies, 23 data points)			
	Mortality	**3 Studies (3 data points)** Garfinkel *et al.*,^83^ 2007; Kearney *et al.*,^65^ 2023; Caravaca *et al.*,^55^ 2018*	**10 Studies (12 data points)** Tapper *et al.*,^38^ 2022 (IG-Zolpidem and IG-Benzodiazepam); *Kutner et al.*,^70^ *2015; Curtin et.al.*,^69^ *2020*; Malik *et al.*,^47^ 2019; *Etherton-Beer et al.*,^37^ *2023* (IG-Open and IG-Blind); *Potter et al.*,^74^ *2016; Bogaerts et al.*,^76^ *2024;* Niznik *et al.*,^44^ 2022; Hayes *et al.*,^48^ 2023; Ruderman *et al.*,^54^ 2018	**3 Studies (3 data points)** Chin-Yee *et al.*,^50^ 2022; Brunetti *et al.*,^45^ 2024; Czikk *et al.*,^53^ 2022*,^k^
	Survival time	**1 Study (1 data point)** *Bogaerts et al.*,^76^ 2024*	**2 Study (2 data points)** Kearney *et al.*,^65^ 2023; Tapper et al. 38, 2022 (IG-Benzodiazepam)*	**2 Studies (2 data points)** Brunetti *et al.*,^45^ 2024*; Tapper *et al.*,^38^ 2022 (IG-Zolpidem)*
	Quality of life outcome (9 studies, 19 data points)			
	General Health-Related Quality of Life (HRQoL)	**1 Study (1 data point)** *Kutner et al.*,^70^ *2015* (McGill Qol score)	**5 Studies (7 data points)** Hurley *et al.*,^35^ 2024b (EQ-5D-5L score and EQ-5D-5L VAS score); *Frankenthal et al.*,^84^ *2014* (SF-12); *Curtin et.al.*,^69^ *2020* (ICECAP-O score); *Etherton-Beer et al.*,^37^ *2023* (IG-Open and IG-Blind EQ-5D-5L); *Potter et al.*,^74^ *2016* (EQ-5D)	-
	Dementia-Specific Quality of life	-	**4 Study (5 data points)** *Bergh et al.*,^72^ *2012* (QoL-ADS, patients’ rating and caregivers’ rating); *Potter et al.*,^74^ *2016* (QOLAD); *Bogaerts et al.*,^76^ *2024* (QUALIDEM); *Curtin et.al*,^69^ *2020* (QUALIDEM)	**-**
	Caregiver-related quality of life	-	**1 Study (2 data points)** *Bogaerts et al.*,^76^ *2024* (CarerQol-7D and CarerQol-7D-VAS)	-
	Symptom and Discomfort	-	**2 Study (3 data points)** *Kutner et al.*,^70^ *2015* (Edmonton Symptom Assessment System scores); Daiello *et al.*,^46^ 2009 (Bowel and Bladder continence)	**1 Study (1 data point)** *Bogaerts et al.*,^76^ *2024* (DS-DAT)
	Clinical events and complication outcomes (13 studies, 37 data points)			
	Acute adverse events	**2 Studies (2 data points)** Chin-Yee *et al.*,^50^ 2022 (bleeding event^n^); Hayes *et al.*,^48^ 2023 (Major bleeding events)	**5 Studies (7 data points)** Tapper *et al.*,^38^ 2022 (IG-Zolpidem and IG-Banzodiazepam: Intracranial Haemorrhage proportion); Brunetti *et al.*,^45^ 2024 (SSE and MB/CRNB); Chin-Yee *et al.*,^50^ 2022 (Thrombotic event^l^); Riveras et al.,^49^ 2024 (Major bleeding or VTE); Hayes *et al.*,^48^ 2023 (Thrombotic event^m^)	**1 Study (1 data point)** *Kutner et al.*,^70^ 2015* (Cardiovascular events)
	Progressive Clinical Deterioration	**-**	**7 Studies (16 data points)** Tapper *et al.*,^38^ 2022 (IG-Zolpidem and IG-Benzodiazepam: Hepatic encephalopathy, IG-Zolpidem and IG-Benzodiazepam: patients with ascites); *Bogaerts et al.*,^76^ *2024* (Change in systolic and diastolic blood pressure); Czikk *et al.*,^53^ 2022 (Serum calcium level); Caravaca *et al.*,^55^ 2018 (Reduction in GFR); *Potter et al.*,^74^ *2016* (Bowel function); *Ruderman et al.*,^54^ *2018* (Serum - phosphate level, albumin level, CRP level, ferritin level, haemoglobin level, bicarbonate level, 25-hydroxy vitamin D level)	**2 Studies (5 data points)** Czikk *et al.*,^53^ 2022 (Serum - Phosphate level, magnesium level); *Ruderman et al.*,^54^ *2018* (Serum - PTH level, calcium level, alkaline phosphatase level)
	Treatment Escalation Requirements	**1 Study (1 data point)** Niznik *et al.*,^39^ 2020a (Need for antipsychotic prescribing)	**3 Study (4 data points)** Ruderman *et al.*,^54^ 2018 (Patient with parathyroidectomy and calciphylaxis); *Bogaerts et al.*,^76^ *2024 (changes in psychotropic drug use); Bergh et al.*,^72^ *2012 (changes in psychotropic drug taken)*	**1 Study (1 data point)** *Caravaca et al.*,^55^ 2018* (Need for dialysis)
Medication-related outcomes		Medication reduction (17 studies, 24 data points)		
	Reduction in total medications number	**15 Studies (15 data points)** Hurley *et al.*,^35^ 2024b; Brunet et al. 57, 2014; *Frankenthal et al.*,^73^ *2017; Kutner et al.*,^70^ *2015; Potter et al.*,^74^ *2016*; Whitty *et al.*,^80^ 2018; *Ferro-Uriguen et al.*,^78^ *2023* (T1 Patients); Saad *et al.*,^59^ 2012*; Whitman *et al.*,^58^ 2018*; Wauters *et al.*,^61^ 2021*,^c^; Suzuki *et al.*,^81^ 2023*; Shirley *et al.*,^66^ 2021*; Poudel *et al.*,^60^ 2015*; Pruskowski *et al.*,^67^ 2017*; McIntyre *et al.*,^68^ 2017*	**3 Studies (5 data points)** *Ferro-Uriguen et al.*,^78^ *2023* (T2 Patients on total medicine number, T1 and T2 patients - hyperpolypharmacy (>10 medicines)); Choukroun *et al.*^64^; 2020, Kearney *et al.*,^65^ 2023	-
	Medication burden and complexity	**2 Studies (2 data points)** Hurley *et al.*,^35^ 2024b (DBI); *Ferro-Uriguen et al.*,^78^ *2023* (T1 Patients : MRCI)	**1 Study (3 data points)** *Ferro-Uriguen et al.*,^78^ *2023* (T1 and T2 Patients – DBI, T2 Patients - MRCI)	-
	Medication appropriateness (10 studies, 15 data points)			
	Modified medication appropriateness index	**1 Study (1 data point)** Hurley *et al.*,^35^ 2024b	-	-
	Reduction in inappropriate medication	**8 Studies (8 data points)** *Frankenthal et al.*,^73^ *2017*; Suhrie *et al.*,^62^ 2009; *Ferro-Uriguen et al.*,^78^ *2023* (T1 Patients); Choukroun *et al.*,^64^ 2020 (Laroche criteria); Gerardi *et al.*,^82^ 2022*; Whitman *et al.*,^58^ 2018*; Wauters *et al.*,^61^ 2021*,^d^; M Chess-Williams *et al.*,^79^ 2024*	**2 Study (2 data points)** *Ferro-Uriguen et al.*,^78^ *2023* (T2 Patients); Choukroun *et al.*,^64^ 2020 (STOPP Criteria)	-
	Number of Potential prescription omissions	**1 Study (1 data point)** Choukroun *et al.*,^64^ 2020	**1 Study (1 data point)** *Frankenthal et al.*,^73^ *2017*	-
	Cognitive burden of anticholinergic medication	**2 Studies (2 data points)** Hurley *et al.*,^35^ 2024b (Anticholinergic Cognitive Burden); Yeh *et al.*,^52^ 2013 (Clinician-rate anticholinergic score)		
	Adverse drug events (6 studies, 9 data points)			
	Adverse events^e^ frequency	**3 Study (3 data points)** Choukroun *et al.*,^64^ 2020; Suzuki et al.,^81^ 2023*,^a^ ; Whitman *et al.*,^58^ 2018*,^b^	**3 Studies (4 data points)** *Etherton-Beer et al.*,^37^ *2023* (IG-Open and IG-Blind); *Kutner et al*,^70^ *2015; Bogaerts et al.*,^76^ *2024*	-
	Severity of adverse events^e^	-	**1 Study (2 data points)** *Etherton-Beer et al.*,^37^ *2023* (IG-Open and IG-Blind)	-
	Drug-Drug Interaction **(1 study 2 data points)**	-	**1 Study (2 data points)** *Ferro-Uriguen et al.*,^78^ *2023* (T1 and T2 Patients)	-
System-related outcomes		Healthcare Expense (13 studies, 13 data points)		
	Reduction in medication cost	**9 Studies (9 data points)** *Curtin et.al.*,^69^ *2020; Frankenthal et al.*,^73^ *2017; Ferro-Uriguen et al.*,^78^ *2023* (T1 Patients); Basri *et al.*,^56^ 2018*; Hurley *et al.*,^36^ 2024a*; Kutner *et al.*,^70^ 2015*; Okafor *et al.*,^77^ 2024; Whitty *et al.*,^80^ 2018*; Kearney *et al.*,^65^ 2023*	**2 Studies (2 data points)** Hurley *et al.*,^35^ 2024b; *Ferro-Uriguen et al.*,^78^ *2023* (T2 Patients)	-
	Reduction in healthcare cost	**1 Study (1 data point)** Whitman *et al.*,^58^ 2018*,^j^	**1 Study (1 data point)** Nakagaito *et al.*,^51^ 2024^f^	-
	Patient Satisfaction (4 studies, 4 data points)	**3 Studies (3 data points)** Gerardi *et al.*,^82^ 2022*,^g^; Shirley et al.,^66^ 2021*,^h^; *Mcntyre et al.*,^68^ 2017*,^i^	**1 Study (1 data point)** *Kutner et al.*,^70^ *2015*	**-**
	Healthcare utilisation (13 studies, 22 data points)			
	Emergency visit	**-**	**2 Studies (2 data points)** *Curtin et.al.*,^69^ *2020;* Hurley *et al.*,^35^ 2024b	-
	Hospital Admission	**2 Studies (3 data points)** Kearney *et al.*,^65^ 2023 (length of hospitalisation and patient number for hospitalisation); Hurley *et al.*,^35^ 2024b*	**6 Studies (8 data points)** *Curtin et.al.*,^69^ *2020; Frankenthal et al.*,^73^ *2017*; Tapper *et al.*,^38^ 2022 (IG-Zolpidem and IG-Benzodiazepam); *Potter et al.*,^74^ *2016;* Nakagaito *et al.*,^51^ 2024 (All cause-hospitalisation proportion); *Etherton-Beer et al.*,^37^ *2023* (IG-Open* and IG-Blind*)	**3 Studies (3 data points)** Nakagaito *et al.*,^51^ 2024 (All cause hospitalisation events per year), Malik *et al.*,^47^ 2019, *Bogaerts et al.*,^76^ *2024*
	Emergency department visit or hospitalisation	-	**1 Study (1 data point)** Niznik *et al.*,^44^ 2022	-
	General Physician consultation	-	**2 Studies (2 data points)** *Curtin et.al.*,^69^ *2020; Potter et al.*,^74^ *2016*	-
	Referral rate	**1 Study (1 data point)** Garfinkel *et al.*,^83^ 2007	-	-
	Length of Hospital Stay	-	**1 Study (1 data point)** Malik *et al.*,^47^ 2019	-
	**All-cause negative events (emergency visit, hospitalisation and mortality)**	-	**1 Study (1 data point)** Niznik *et al.*,^40^ 2020b	-

PTH: parathyroid hormone; ADL: activities of daily living; NPI-Q: neuropsychiatric inventory questionnaire; NPI: neuro-psychiatric inventory; MMSE: mini-mental state examination; UPDRS: united Parkinson’s disease rating scale; ICECAP-O: ICEpop CAPability measure for older people; Qualidem: indicates dementia-specific quality of life assessment scale; SF-12: quality of life using medical outcomes study 12-item short-form health survey; SSE: stroke or systemic embolism; MB/CRNMB: major or clinically relevant non-major bleeding; NPI-NH: neuropsychiatric index – nursing home version; MBI: modified Barthel index; QOLAD: quality of life in Alzheimer’s dementia; QoL-ADS: quality of life-Alzheimer’s disease scale; PQSI: Pittsburgh sleep quality index; EQ-5D-5L: EuroQol 5-dimension 5-level questionnaires; EQ-5D-5L VAS score: EuroQol 5-dimension 5-level visual analogue scale (VAS); MDS-CPS: minimum data set cognitive performance scale; CAM: confusion assessment method; AES-10: apathy evaluation scale-10; CDS: care dependency scale; CarerQoL-7D: care-related quality-of-life-7 dimension; CarerQol-7D VAS: care-related quality of life-7 dimensions visual analogue scale; DS-DAT: discomfort scale for patients with dementia of the Alzheimer type; VTE: venous thromboembolism; GFR: glomerular filtration rate; T1: dementia-like trajectory patients; T2: end stage organ failure trajectory; DBI: drug burden index; MRCI: medication regimen complexity index; ADE: adverse drug events; SAE: serious adverse events; DDIs: drug-drug interactions; PSQI: Pittsburgh sleep quality index; PIP: potentially inappropriate prescription; PPO: potential prescription omission.

a 44.7% (55/123) symptoms improved.

b Eighteen out of 29 were available for follow up, where 16 patients previously reported symptoms or side effects reported reduction on it.

c Total medication decreased by 35%.

d At least one PIM reduction in 25.9%.

e Adverse events, medication side effects.

f Healthcare costs refers to in-hospital medical costs and cost for sodium–glucose cotransporter 2 inhibitors (SGLT2i) in this study.

g One of twenty-nine patient expressed transient dissatisfaction due to return of symptom.

h Fifty-six percent (14/25) of responded satisfied.

i No patient reported any concerns (assessed based on symptom management).

j Healthcare expenditure were calculated using standardised cost avoidance values from the University Health System Consortium (UHC), including prevention of minor and major adverse events, medication teaching, and detailed medication history.

k One (out of 29) died from GI bleeding after 2 days of PPIs switched to every second day dosing.

l Hospital admission or emergency department visit with ischaemic stroke, transient ischaemic attack or venous thromboembolism.

m Thrombotic events indicates acute myocardial infarction, systemic embolism, venous thromboembolism, ischaemic stroke, and transient ischaemic at-tack.

n Hospital admission or emergency department visit with intracranial, gastrointestinal (upper or lower) or other (primarily genitourinary and respiratory) bleeding.

### Clinical-related outcomes

***Cognitive and neuropsychiatric outcomes:*** Most studies reporting cognitive and neuropsychiatric outcomes found no significant changes after deprescribing.^37,46,52,63,71,72,74–76^ However, two studies observed notable effects. Bogaerts *et al.*^76^ reported the significant worsening of neuropsychiatric symptoms and subsequent increased distress after the deprescribing. Etherton-Beer *et al.*^37^ assessed the impact of deprescribing in blind and open intervention groups and found significant worsening effects in the blind group but no significant change in the open intervention group (Table 3).

***Functional and physical health outcomes:*** The majority of studies found that deprescribing had no significant impact on functional and physical health.^37,38,46,52,54,69–72,74,76,84^ Three studies (two^38,40^ with statistical significance and one^35^ without statistical analysis) reported deprescribing reduced falls, while one study^76^ reported that deprescribing significantly increased the risk of falls. Tapper *et al.*^38^ (for zolpidem deprescribing) and Etherton-Beer *et al.*^37^ showed deprescribing reduced the number of fractures, while no studies reported that deprescribing increased fracture risk. On functional independence, no studies reported that deprescribing improved level of function, while eight studies showed deprescribing had no significant effect on function.^37,46,52,70,72–74,76^ One study (Daiello *et al.*^46^) showed that deprescribing significantly reduced the time patients spent in leisure activities after cholinesterase inhibitors were reduced/stopped in dementia nursing home residents (Table 3).

***Behavioural and mood outcomes:*** The five studies reporting on these outcomes did not find evidence that deprescribing improved behavioural and mood related outcomes. For example, Daiello *et al.*^46^ reported significant worsening of behavioural symptoms but no significant changes in depression rating scale due to deprescribing. Whereas Bergh *et al.*^72^ reported progression of depressive symptom by deprescribing. On sleep quality, Ruths *et al.*^75^ reported a significant reduction in sleep efficiency after deprescribing but no significant changes in overall day- and night-time activities. Potter *et al.*^74^ and Niznik *et al.*^39^ reported that deprescribing had no significant effect on sleep quality and aggressive behaviour (Table 3).

***Mortality and survival outcomes:*** The majority of the studies (*n* = 10) showed that deprescribing did not significantly worsen mortality,^37,38,44,47,48,54,69,70,74,76^ while three studies showed that deprescribing decreased mortality – two with statistical significance^65,83^ and one^55^ without statistical analysis. Alternatively, Brunetti *et al.*^45^ and Chin-Yee *et al.*^50^ reported significantly increased mortality after deprescribing, and Czikk *et al.*^53^ reported the death of one participant 2 days after the deprescribing of a PPI (the study authors suggest the cause of death was due to a bleed). Among the four studies that reported outcomes on survival without performing statistical analysis for significance, two studies (Brunetti *et al.*^45^ and Tapper *et al.*^38^ for zolpidem deprescribing) reported that survival time decreased after deprescribing (Table 3).

***Quality of life related outcomes:*** One study (Kutner *et al.*^70^) showed deprescribing significantly improved quality of life outcomes, while all other studies reported deprescribing had no significant effect on general health-related quality of life.^35,37,69,74,84^ Studies reporting on dementia-specific quality of life^69,72,74,76^ and caregiver-related quality of life^76^ showed deprescribing did not significantly impact these outcomes. On symptoms and discomfort, Bogaerts *et al.*^76^ reported that deprescribing significantly worsened these outcomes, while two other studies (Kutner *et al.*^70^ and Daiello *et al.*^46^) reported no significant difference (Table 3).

***Clinical events and complication outcome:*** The majority of studies reporting acute adverse events related outcomes found that deprescribing did not significantly change these outcomes.^38,45,48–50^ However, two studies (Chin-Yee *et al.*^50^ and Hayes *et al.*^48^) did report significant improvement in acute adverse events (e.g. bleeding events), while one study (Kutner *et al.*^70^) reported that deprescribing was associated with higher comparative cardiovascular events – although this was not supported with conducting statistical analysis. There were no studies that reported improvements in progressive clinical parameters with deprescribing interventions. However, there was two studies that reported a significant deterioration in specific metabolic parameters following deprescribing (e.g. changes in serum levels of phosphate, magnesium).^53,54^ Niznik *et al.*^39^ found that deprescribing cholinesterase inhibitors in dementia patients was not linked to clinical deterioration; rather, it significantly reduced likelihood of initiating antipsychotic medications. On contrast, Bogaerts *et al.*^76^ and Bergh *et al.*^72^ found no significant difference in psychotropic medicine usage following the deprescribing of antihypertensive and antidepressants, respectively. Caravaca *et al.*^55^ noted deprescribing increased dialysis needs but they did not perform any statistical analysis to support this observation (Table 3).

### Medication-related outcomes

***Medication reduction:*** The majority of studies (*n* = 15) reported that deprescribing reduced the total number of medications used by patients. Most of the studies reported these finding with statistical signficance,^35,57,70,73,74,78,80^ while some of the studies reported reduction without performing a statistical analysis for significance.^58–61,66,[Bibr bibr67-02692163261416281]–68,81^ Notably, no studies reported an increase in the total number of medications after deprescribing.

Two studies reported medication burden and medication complexity outcomes. Hurley *et al.*^35^ assessed the Drug Burden Index (DBI) and found deprescribing significantly reduced DBI scores (meaning the medication burden was reduced for patients), while another study, by Ferro-Uriguen *et al.*,^78^ found deprescribing did not significantly change DBI scores (Table 3).

***Medication appropriateness:*** The majority of studies reported deprescribing reduced the prescribing of inappropriate medication (four studies^62,64,73,78^ were with statistical significance and four ^58,61,79,82^ without statistical analysis). Only two studies (Ferro-Uriguen *et al.* focussing on people with organ failure^78^ and Choukroun *et al.* guided by the STOPP Criteria)^64^ reported deprescribing had no significant impact on reducing inappropriate medication. Two studies that reported on the anticholinergic burden (Hurley *et al.*,^35^ Yeh *et al.*^52^) found a significant reduction due to deprescribing (Table 3).

***Adverse drug events:*** Among seven studies exploring the impact on adverse drug events, three studies^58,64,81^ reported that deprescribing was associated with reduced adverse drug events, while two studies^58,81^ did not report level of statistical significance and did not include a control group. The other four studies^37,70,72,76^ found that deprescribing did not significantly change adverse drug events. Only one study^78^ explored how deprescribing impacts reported drug-drug interactions and found no significant difference (Table 3).

### System-related outcomes

***Healthcare costs:*** Out of 11 studies reporting on medication costs, 9 studies^36,56,65,69,70,73,77,78,80^ reported that deprescribing reduces costs, where 6 studies^36,56,65,70,77,80^ were without statistical analysis for significance. Only two^35,78^ reported there was no significant difference in medication costs. Two studies reported changes in overall healthcare costs due to deprescribing: Nakagaito *et al.*^51^ reported deprescribing did not significantly reduce healthcare costs, while Whitman *et al.*^58^ reported a healthcare cost reduction without performing statistical analysis for significance. No studies reported that deprescribing increases overall healthcare or medication costs (Table 3).

***Patient satisfaction:*** Four studies reported on patient satisfaction outcomes, and none of them showed deprescribing reduced patient satisfaction scores. Specifically, Gerardi *et al.*,^82^ McIntyre *et al.*^68^ and Shirley *et al.*^66^ reported that deprescribing was associated with improved patient satisfaction scores, while Kutner *et al.*^70^ reported no significant difference in scores (Table 3).

***Healthcare utilisation:*** Hurley *et al.*^35^ and Curtin *et al.*^69^ reported no statistically significant differences in emergency department utilisation following deprescribing interventions. Similarly, most studies reported no significant difference in hospital admission burden.^38,51,69,73,74^ However, three studies reported that deprescribing significantly increased the likelihood for hospital admission,^47,51,76^ while two studies^35,65^ reported that deprescribing decreased the likelihood – where one study^35^ was without statistical analysis. Malik *et al.*,^47^ Niznik *et al.*^40^ and Niznik *et al.*^44^ explored the length of hospital stay, combined negative events (emergency visits, hospitalisation and mortality), and either emergency visits or hospitalisation, respectively. The authors for these studies reported that deprescribing did not significantly affect any of these outcomes. Garfinkel *et al.*^83^ explored the referral rate to acute care facilities and found that deprescribing significantly reduced the rate of referral. No significant effect of deprescribing was reported in physician consultations rate, as reported by two studies^69,74^ (Table 3).

### Critical appraisal of included studies

For randomised controlled studies, blindness to participants and those delivering the intervention were common concerns. Non-similar study groups, identification and addressing compounding factors, and follow-up related information were the major non-complying criteria for cohort studies. A common factor identified in most quasi-experimental studies was having a single arm without comparison groups. The detailed scoring and overall rating of each study are available in the Supplemental file (Supplemental Tables 11–15).

## Discussion

In this systematic review, we have comprehensively summarised deprescribing outcomes for people with life-limiting conditions and organised these outcomes into three broad groups representing clinical, medication and system-level domains.

Our review highlights several key findings with implications for patients, healthcare professionals and policymakers. The evidence shows that, for people with life-limiting conditions, deprescribing can improve medication-related outcomes: for example, deprescribing can reduce the total medication number taken by patients, as well as improving medication appropriateness. There was strong evidence to support this, and this finding corresponds to previous reviews: Shrestha *et al.*^33^ reported that deprescribing reduces the number of medications and improves appropriateness of medication in people with life limiting illness and limited life expectancy. A recent 2025 systematic review and network meta-analysis among older adults with chronic diseases also confirmed that deprescribing is an appropriate intervention for reducing inappropriate prescribing.^85^ Similar findings of medication reduction have also been reported by two umbrella reviews on deprescribing interventions in older people.^27,29^ While it is perhaps not surprising that deprescribing approaches reduce the total number of medication used by patients, the reduction of inappropriate medication is less predictable, but importantly, this is essential to reduce potential adverse effects from medication. In view of these findings, healthcare professionals whose responsibilities include prescription and review of medication for people with life limiting illnesses should initiate deprescribing to reduce the medication count and improve prescribing appropriateness, whilst carefully monitoring the effects.

The impact of deprescribing on the other outcomes was found to be more complex. For clinical outcomes, evidence suggests that deprescribing does not necessarily lead to improvement, but in most cases, it does not result in harm. For example, for studies reporting on mortality (16 studies), the majority of studies (10 studies) reported that deprescribing had no impact; 3 studies reported deprescribing increased mortality, while 3 reported deprescribing reduced mortality.^31^ In line with this, a recent 2024 umbrella review on deprescribing interventions in older adults by Chua *et al.*,^29^ which reported that 17 out of 19 systematic reviews found no significant impact on mortality outcomes with none of the reviews reporting increased mortality. This suggests that while there is a higher likelihood of no effects on mortality and survival time, there is a possibility that deprescribing can increase mortality in people with life-limiting illness in certain contexts. Understanding the factors influencing the risk of mortality in life-limiting conditions is complex due to the involvement of multifaceted aspects, including physiological changes associated with the progression of life-limiting conditions.^26,86,87^ Mostly neutral findings were also found for the behavioural and mood outcomes and the functional and physical health outcomes. Therefore, when considering deprescribing in relation to health-related outcomes, it is essential to carefully assess the patient’s underlying conditions, the specific medications targeted for deprescribing, and to ensure ongoing monitoring to maintain safety.

Literatures generally reported beneficial effects like medication-related domains in most system-related outcomes. The evidence from the included studies indicated that deprescribing consistently reduced medication cost, which is an expected outcome. However, the evidence on overall healthcare cost reduction was limited, with one reported beneficial and another no significant difference. This might be linked with the differences in their methodology applied for healthcare cost estimation: Whitman *et al*.^58^ modelled cost assumptions associated with adverse events prevention and pharmacist interventions, and did not assess with statistical analysis of significance, whereas Nakagaito *et al.*^51^ calculated actual hospitalisation and medication costs and assessed difference with statistical analysis of significance. Notably, none of them accounted for the cost associated with long-term health consequences of deprescribing. Furthermore, external evidence (Wojtowycz *et al.*^88^) suggests that the overall healthcare cost could be increased in some cases, such as when new medications are added after deprescribing, highlighting a critical and unexplored areas. Therefore, further studies are essential to explore these nuances. Followingly, deprescribing approach did not appear to adversely affect patient satisfaction. However, healthcare utilisation outcomes were largely unchanged, indicating for further research to clarify whether specific patients population and deprescribed medication are more associated with adverse events for re-admission. To summarise the system-related outcomes, the majority studies showed that deprescribing generally had no negative effects.

The neutrality reported across most outcomes underscore the complexity of measuring the impact of deprescribing. While the absence of harmful effects in the majority of studies is encouraging, the predominantly neutral effects raise a critical question on whether deprescribing retains modest-benefit only or current outcome measures are insufficient to capture meaningful changes or make appropriate conclusions. Further research is needed to elucidate the specific factors that drive variations in deprescribing outcomes. Additionally, some reported negative effects on some outcome variables highlight the need for a careful monitoring and individualised patient-centred approaches to deprescribing. A recent qualitative study highlighted that patient wanted to have a voice in deprescribing decision-making, but at present this does not always occur.^89^

### Strength and limitations

A key strength of this review is its comprehensive synthesis of deprescribing outcome across a broad spectrum of life-limiting conditions, which identified a larger body of evidence (46 studies) compared to previous similar review by Shrestha et al.^33^ in 2020 (9 studies). While Shrestha *et al.*^33^ focussed on older adults (>65) with a short life expectancy (up to 2 years), our pragmatic approach draws the evidence from a wider range of serious illnesses, reflecting the clinical reality of life-limiting conditions where age and life expectancy are often uncertain. However, this fundamental difference precludes a direct quantitative comparison of findings but demonstrates the relevance of deprescribing to a wider palliative care context.

The study does, however, have few limitations that should be acknowledged. First, the included studies were heterogenous in terms of study design, settings, outcome assessment and reporting, selection and deprescribing of medication that limited to conduct meta-analysis. Similarly, this study did not include those studies which were not published or available in English language, not peer reviewed, published before 2000, and did not provide sufficient information to conclude that their study population had life-limiting conditions.

Furthermore, while this systematic review included 46 original studies there are a number of important evidence gaps. First, the included studies were mostly conducted in North America, Europe and Australia. There were few representations from Asia and no representation from other continents. It is not clear if – or how – the healthcare system in which the deprescribing takes place impacts on the deprescribing outcome. Second, there were variations in how the deprescribing process was undertaken. Some studies directly targeted specific medications, while other studies have reported deprescribing a range of medications with the help of various tools and clinicians’ recommendations. The deprescribing duration, success or failure rate of deprescribing, and incorporation of other medication-related interventions (e.g. medication review, medication reconciliation, adjustment in medicine and their dosage) were not consistently reported across the studies. Finally, patients in the included studies had a variety of life-limiting conditions, with dementia and cancer being the most common reflecting the diverse disease trajectories being considered.

### Future directions

To strengthen the literature to further support evidence-based deprescribing, future studies could explore the impact of the deprescribing of specific medication classes in specific patient and disease contexts. Similarly, future studies should clearly define the success rate of deprescribing, including reporting data on whether the deprescribed medication remained stopped or reduced or restarted. Improving the consistency of deprescribing reporting across studies should further strengthen the evidence on deprescribing outcomes. Future studies could further investigate the factors influencing successful deprescribing to inform the development of future deprescribing interventions in people with life-limiting conditions.

Moreover, the number of studies contributing to each outcome variable was not uniform. While medication reduction, healthcare utilisation and mortality were well reported, patient-centred outcomes (such as quality of life, patient satisfaction), long-term safety endpoints (such as specific clinical complications, adverse drug events), and overall healthcare cost were reported by small number of studies. Thus, these gaps should be prioritised in future studies.

## Conclusion

This systematic review suggests that deprescribing approach offers several benefits, including reduced medication burden and costs in people with LLCs. While there is no strong evidence for harm, a small proportion of patients may face risks, so careful monitoring is essential. Further studies exploring deprescribing interventions specific to patients’ disease conditions are warranted to strengthen the evidence on deprescribing outcomes.

## Supplemental Material

sj-docx-1-pmj-10.1177_02692163261416281 – Supplemental material for Outcomes of deprescribing for people with life-limiting conditions: A systematic review Supplemental material, sj-docx-1-pmj-10.1177_02692163261416281 for Outcomes of deprescribing for people with life-limiting conditions: A systematic review by Rajeev Shrestha, Emily Shaw, Liam Mullen, David Sinclair, Felicity Dewhurst and Adam Todd in Palliative Medicine

## References

[1] Palliative Care Dictionary. What is life-limiting condition - meaning and definition - Pallipedia, https://pallipedia.org/life-limiting-condition/ (accessed 8 May 2025).

[2] Mack DS Tjia J Lapane KL. Defining life-limiting illness in the nursing home population: identifying a population to benefit from palliative care services. J Nurs Home Res Sci 2022; 8: 1–5. DOI: 10.14283/jnhrs.2022.1

[3] Cadogan CA Murphy M Boland M , et al. Prescribing practices, patterns, and potential harms in patients receiving palliative care: a systematic scoping review. Explor Res Clin Soc Pharm 2021; 3: 100050. 35480601 10.1016/j.rcsop.2021.100050 PMC9031741

[4] Chen LJ Trares K Laetsch DC , et al. Systematic review and meta-analysis on the associations of polypharmacy and potentially inappropriate medication with adverse outcomes in older cancer patients. J Gerontol A 2021; 76: 1044–1052. 10.1093/gerona/glaa128 32459845

[5] Kim S Lee H Park J , et al. Global and regional prevalence of polypharmacy and related factors, 1997-2022: an umbrella review. Arch Gerontol Geriatr 2024; 124: 105465. 38733922 10.1016/j.archger.2024.105465

[6] Delara M Murray L Jafari B , et al. Prevalence and factors associated with polypharmacy: a systematic review and meta-analysis. BMC Geriatr 2022; 22(1): 601. 35854209 10.1186/s12877-022-03279-x PMC9297624

[7] McNeil MJ Kamal AH Kutner JS , et al. The burden of polypharmacy in patients near the end of life. J Pain Symptom Manag 2016; 51: 178–183.e2. 10.1016/j.jpainsymman.2015.09.003 PMC4733587 26432571

[8] Masumoto S Hosoi T Nakamura T , et al. Polypharmacy and potentially inappropriate medications in patients with advanced cancer: prevalence and associated factors at the end of life. J Palliat Med 2024; 27: 749–755. 38354283 10.1089/jpm.2023.0520

[9] Ramsdale E Mohamed M Yu V , et al. Polypharmacy, potentially inappropriate medications, and drug-drug interactions in vulnerable older adults with advanced cancer initiating cancer treatment. Oncologist 2022; 27: e580–e588. 10.1093/oncolo/oyac053 PMC9255971 35348764

[10] Jaramillo-Hidalgo J Lozano-Montoya I Tornero-Torres O , et al. Prevalence of potentially inappropriate prescription in community-dwelling patients with advanced dementia and palliative care needs. Rev Esp Geriatr Gerontol 2021; 56: 203–207. 34001344 10.1016/j.regg.2021.03.001

[11] Disalvo D Luckett T Luscombe G , et al. Potentially Inappropriate prescribing in Australian nursing home residents with advanced dementia: a substudy of the IDEAL study. J Palliat Med 2018; 21: 1472–1479. 30106321 10.1089/jpm.2018.0070

[12] Riedl L Kiesel E Hartmann J , et al. A bitter pill to swallow - polypharmacy and psychotropic treatment in people with advanced dementia. BMC Geriatr 2022; 22: 214. 35296254 10.1186/s12877-022-02914-x PMC8925050

[13] Kirci O Cubukcu M Bahsi R , et al. Examining potentially inappropriate medication use among elderly individuals in palliative care: a comprehensive study. Heliyon 2024; 10: e30635. 10.1016/j.heliyon.2024.e30635 PMC11108814 38778926

[14] Granger BB Tulsky JA Kaufman BG , et al. Polypharmacy in palliative care for advanced heart failure: the PAL-HF experience. J Card Fail 2022; 28: 334–338. 34628013 10.1016/j.cardfail.2021.08.021 PMC8898052

[15] Scullion L Dodds H Liu Q , et al. Medication use in the last year of life: a cross-sectional hospice study. BMJ Support Palliat Care 2022; 12: e740–e743. 10.1136/bmjspcare-2019-002101 32788273

[16] Alwidyan T McCorry NK Black C , et al. Prescribing and deprescribing in older people with life-limiting illnesses receiving hospice care at the end of life: a longitudinal, retrospective cohort study. Palliat Med 2024; 38: 121–130. 38032069 10.1177/02692163231209024 PMC10798021

[17] Rojas-Solé C Pinilla-González V Lillo-Moya J , et al. Integrated approach to reducing polypharmacy in older people: exploring the role of oxidative stress and antioxidant potential therapy. Redox Rep 2024; 29: 2289740. 10.1080/13510002.2023.2289740 PMC10732214 38108325

[18] Malakouti SK Javan-Noughabi J Yousefzadeh N , et al. A systematic review of potentially inappropriate medications use and related costs among the elderly. Value Health Reg Issues 2021; 25: 172–179. 34311335 10.1016/j.vhri.2021.05.003

[19] Fitzpatrick D Gallagher PF. Polypharmacy: definition, epidemiology, consequences and solutions. Pract Iss Geriatr 2023; Part F12: 15–31.

[20] G N S Reddy R V V D , et al. A study of polypharmacy and its consequences in geriatric patients. JCPP 2024; 4: 1–17.

[21] Mangin D Bahat G Golomb BA , et al. International group for reducing inappropriate medication use & polypharmacy (IGRIMUP): position statement and 10 recommendations for action. Drugs Aging 2018; 35: 575–587. 30006810 10.1007/s40266-018-0554-2 PMC6061397

[22] Murali KP Merriman JD Yu G , et al. Complex care needs at the end of life for seriously ill adults with multiple chronic conditions. J Hosp Palliat Nurs 2023; 25: 146–155. 37040386 10.1097/NJH.0000000000000946 PMC10175220

[23] Pierotti D Greenberg EL. It’s time to talk: starting a conversation about discontinuing medications at end of life. Home Healthc Now 2016; 34: 228–229. 27023303 10.1097/NHH.0000000000000368

[24] Lo UC Musa H Li J , et al. Patient beliefs associated with medication hesitancy in palliative care: a systematic review using the theory of planned behavior. Palliat Support Care 2024; 22: 610–622. 36503650 10.1017/S1478951522001547

[25] Ramsburg H Moriarty HJ MacKenzie Greenle M. End-of-life symptoms in adult patients with stroke in the last two years of life: an integrative review. Am J Hosp Palliat Care 2024; 41: 831–839. 37615127 10.1177/10499091231197657

[26] Cabrera JA Mota M Pais C , et al. Deprescription in palliative care. Cureus 2023; 15: e39578. 10.7759/cureus.39578 PMC10292863 37378207

[27] Veronese N Gallo U Boccardi V , et al. Efficacy of deprescribing on health outcomes: an umbrella review of systematic reviews with meta-analysis of randomized controlled trials. Ageing Res Rev 2024; 95: 102237. 38367812 10.1016/j.arr.2024.102237

[28] Kornholt J Bülow C Sørensen AMS , et al. Scoping review of randomized trials with discontinuation of medicines in older adults. J Am Med Dir Assoc 2022; 23: 1926.e11–1926.e35. 10.1016/j.jamda.2022.06.010 35850165

[29] Chua S Todd A Reeve E , et al. Deprescribing interventions in older adults: an overview of systematic reviews. PLoS One 2024; 19: e0305215. 10.1371/journal.pone.0305215 PMC11182547 38885276

[30] Quek HW Page A Lee K , et al. The effect of deprescribing interventions on mortality and health outcomes in older people: an updated systematic review and meta-analysis. Br J Clin Pharmacol 2024; 90: 2409–2482. 39164070 10.1111/bcp.16200

[31] Shrestha S Poudel A Cardona M , et al. Impact of deprescribing dual-purpose medications on patient-related outcomes for older adults near end-of-life: a systematic review and meta-analysis. Ther Adv Drug Saf 2021; 12: 20420986211052344. 10.1177/20420986211052343 PMC8543710 34707802

[32] Cardona M Stehlik P Fawzy P , et al. Effectiveness and sustainability of deprescribing for hospitalized older patients near end of life: a systematic review. Expert Opin Drug Saf 2021; 20: 81–91. 33213216 10.1080/14740338.2021.1853704

[33] Shrestha S Poudel A Steadman K , et al. Outcomes of deprescribing interventions in older patients with life-limiting illness and limited life expectancy: a systematic review. Br J Clin Pharmacol 2020; 86: 1931–1945. 31483057 10.1111/bcp.14113 PMC7495295

[34] Cardona-Morrell M Hillman K. Development of a tool for defining and identifying the dying patient in hospital: criteria for screening and triaging to appropriate alternative care (CriSTAL). BMJ Support Palliat Care 2015; 5: 78–90. 10.1136/bmjspcare-2014-000770 PMC4345773 25613983

[35] Hurley E Dalton K Byrne S , et al. Pharmacist-led deprescribing using STOPPFRAIL for frail older adults in nursing homes. J Am Med Dir Assoc 2024; 25: 105122. 38950585 10.1016/j.jamda.2024.105122

[36] Hurley E Byrne S Walsh E , et al. Cost avoidance of pharmacist-led deprescribing using STOPPFRAIL for older adults in nursing homes. Int J Clin Pharm 2024; 46: 1163–1171. 38967733 10.1007/s11096-024-01749-3 PMC11399282

[37] Etherton-Beer C Page A Naganathan V , et al. Deprescribing to optimise health outcomes for frail older people: a double-blind placebo-controlled randomised controlled trial-outcomes of the Opti-med study. Age Ageing 2023; 52: 1–10. 10.1093/ageing/afad081 PMC10226731 37247404

[38] Tapper EB Zhao Z Winder GS , et al. Deprescribing zolpidem reduces falls and fractures in patients with cirrhosis. JHEP Reports 2022; 4: 100478. 35493764 10.1016/j.jhepr.2022.100478 PMC9052149

[39] Niznik JD Zhao X He M , et al. Impact of deprescribing AChEIs on aggressive behaviors and antipsychotic prescribing. Alzheimer Dement 2020; 16: 630–640. 10.1002/alz.12054 PMC7138718 32052930

[40] Niznik JD Zhao X He M , et al. Risk for health events after deprescribing acetylcholinesterase inhibitors in nursing home residents with severe dementia. J Am Geriatr Soc 2020; 68: 699–707. 31769507 10.1111/jgs.16241 PMC7477721

[41] Aromataris E Lockwood C Porritt K , et al. JBI manual for evidence synthesis. JBI, https://synthesismanual.jbi.global (2024).

[42] Barker TH Habibi N Aromataris E , et al. The revised JBI critical appraisal tool for the assessment of risk of bias for quasi-experimental studies. JBI Evid Synth 2024; 22: 378–388. 38287725 10.11124/JBIES-23-00268

[43] Barker TH Stone JC Sears K , et al. The revised JBI critical appraisal tool for the assessment of risk of bias for randomized controlled trials. JBI Evid Synth 2023; 21: 494–506. 36727247 10.11124/JBIES-22-00430

[44] Niznik JD Zhao X Slieanu F , et al. Effect of deintensifying diabetes medications on negative events in older veteran nursing home residents. Diabetes Care 2022; 45: 1558–1567. 35621712 10.2337/dc21-2116 PMC9274227

[45] Brunetti E Presta R Okoye C , et al. Predictors and outcomes of oral anticoagulant deprescribing in geriatric inpatients with atrial fibrillation: a retrospective multicenter cohort study. J Am Med Dir Assoc 2024; 25: 545–551.e4. 10.1016/j.jamda.2024.01.011 38359897

[46] Daiello LA Ott BR Lapane KL , et al. Effect of discontinuing cholinesterase inhibitor therapy on behavioral and mood symptoms in nursing home patients with dementia. Am J Geriatr Pharmacother 2009; 7: 74–83. 19447360 10.1016/j.amjopharm.2009.04.002

[47] Malik A Masson R Singh S , et al. Digoxin discontinuation and outcomes in patients with heart failure with reduced ejection fraction. J Am Coll Cardiol 2019; 74: 617–627. 31370952 10.1016/j.jacc.2019.05.064 PMC10465068

[48] Hayes KN Zhang T Kim DH , et al. Benefits and harms of standard versus reduced-dose direct oral anticoagulant therapy for older adults with multiple morbidities and atrial fibrillation. J Am Heart Assoc 2023; 12: 29865. 10.1161/JAHA.122.029865 PMC10727413 37929769

[49] Riveras A Crul M van der Kloes J , et al. A tool for deprescribing antithrombotic medication in palliative cancer patients: a retrospective evaluation. J Pain Palliat Care Pharmacother 2024; 38: 20–27. 38109061 10.1080/15360288.2023.2288093

[50] Chin-Yee N Gomes T Tanuseputro P , et al. Anticoagulant use and associated outcomes in older patients receiving home palliative care: a retrospective cohort study. CMAJ 2022; 194: E1198–E1208. 10.1503/cmaj.220919 PMC9477253 36096505

[51] Nakagaito M Imamura T Ushijima R , et al. The impact of the withdrawal of SGLT2 inhibitors on clinical outcomes in patients with heart failure. J Clin Med 2024; 13: 3196. 38892907 10.3390/jcm13113196 PMC11172815

[52] Yeh Y-C Liu C-L Peng L-N , et al. Potential benefits of reducing medication-related anticholinergic burden for demented older adults: a prospective cohort study. Geriatr Gerontol Int 2013; 13: 694–700. 23216534 10.1111/ggi.12000

[53] Czikk D Parpia Y Roberts K , et al. De-prescribing proton pump inhibitors in patients with end stage kidney disease: a quality improvement project. Can J Kidney Health Dis 2022; 9: 20543581221106244. 10.1177/20543581221106244 PMC9243371 35782023

[54] Ruderman I Smith ER Toussaint ND , et al. Longitudinal changes in bone and mineral metabolism after cessation of cinacalcet in dialysis patients with secondary hyperparathyroidism. BMC Nephrol 2018; 19: 113. 29764395 10.1186/s12882-018-0910-9 PMC5952622

[55] Caravaca F Caravaca-Fontán F Azevedo L , et al. Changes in renal function after discontinuation of vitamin D analogues in advanced chronic kidney disease. Nefrol 2018; 38: 179–189. 10.1016/j.nefro.2017.05.012 28676189

[56] Basri DS DiScala SL Brooks AT , et al. Analysis of inpatient hospice pharmacist interventions within a veterans affairs medical center. J Pain Palliat Care Pharmacother 2018; 32: 240–247. 31290723 10.1080/15360288.2019.1615025

[57] Molist Brunet N Sevilla-Sánchez D Amblàs Novellas J , et al. Optimizing drug therapy in patients with advanced dementia: a patient-centered approach. Eur Geriatr Med 2014; 5: 66–71.

[58] Whitman A DeGregory K Morris A , et al. Pharmacist-led medication assessment and deprescribing intervention for older adults with cancer and polypharmacy: a pilot study. Support Care Cancer 2018; 26: 4105–4113. 29869294 10.1007/s00520-018-4281-3 PMC6204077

[59] Saad M Harisingani R Katinas L. Impact of geriatric consultation on the number of medications in hospitalized older patients. Consult Pharm 2012; 27: 42–48. 22231997 10.4140/TCP.n.2012.42

[60] Poudel A Peel NM Mitchell CA , et al. Geriatrician interventions on medication prescribing for frail older people in residential aged care facilities. Clin Interv Aging 2015; 10: 1043–1051. 26150708 10.2147/CIA.S84402 PMC4485794

[61] Wauters M Elseviers M Vander Stichele R , et al. Efficacy, feasibility and acceptability of the OptiMEDs tool for multidisciplinary medication review in nursing homes. Arch Gerontol Geriatr 2021; 95: 104391. 33819776 10.1016/j.archger.2021.104391

[62] Suhrie EM Hanlon JT Jaffe EJ , et al. Impact of a geriatric nursing home palliative care service on unnecessary medication prescribing. Am J Geriatr Pharmacother 2009; 7: 20–25. 19281937 10.1016/j.amjopharm.2009.02.001 PMC4870890

[63] Bravo-José P Sáez-Lleó CI Peris-Martí JF. Deprescribing antipsychotics in long term care patients with dementia. Farm Hosp 2019; 43: 140–145. 31276444 10.7399/fh.11217

[64] Choukroun C Leguelinel-Blache G Roux-Marson C , et al. Impact of a pharmacist and geriatrician medication review on drug-related problems in older outpatients with cancer. J Geriatr Oncol 2021; 12: 57–63. 32800700 10.1016/j.jgo.2020.07.010

[65] Kearney A Tiwari N Cullen O , et al. Improving palliative and supportive care in advanced cirrhosis: the HepatoCare model of integrated collaborative care. Intern Med J 2023; 53: 1963–1971. 37812158 10.1111/imj.16248

[66] Shirley L DiScala S Brooks A , et al. Pilot of a pharmacist-integrated interprofessional team to optimize prescribing in a telemedicine palliative care clinic. JACCP 2021; 4: 1093–1099.

[bibr67-02692163261416281] Pruskowski J Handler SM. The DE-PHARM project: a pharmacist-driven deprescribing initiative in a nursing facility. Consult Pharm 2017; 32: 468–478. 29029668 10.4140/TCP.n.2017.468

[68] McIntyre C McQuillan R Bell C , et al. Targeted deprescribing in an outpatient hemodialysis unit: a quality improvement study to decrease polypharmacy. Am J Kidney Dis 2017; 70: 611–618. 28416321 10.1053/j.ajkd.2017.02.374

[69] Curtin D Jennings E Daunt R , et al. Deprescribing in older people approaching end of life: a randomized controlled trial using STOPPFRAIL criteria. J Am Geriatr Soc 2020; 68: 762–769. 31868920 10.1111/jgs.16278

[70] Kutner JS Blatchford PJ Taylor DH , et al. Safety and benefit of discontinuing statin therapy in the setting of advanced, life-limiting illness: a randomized clinical trial. JAMA Intern Med 2015; 175: 691–700. 25798575 10.1001/jamainternmed.2015.0289 PMC4618294

[71] Tse W Frisina PG Hälbig TD , et al. The effects of withdrawal of dopaminergic medication in nursing home patients with advanced parkinsonism. J Am Med Dir Assoc 2008; 9: 670–675. 18992700 10.1016/j.jamda.2008.07.001

[72] Bergh S Selbæk G Engedal K. Discontinuation of antidepressants in people with dementia and neuropsychiatric symptoms (DESEP study): double blind, randomised, parallel group, placebo controlled trial. BMJ 2012; 344: e1566. 10.1136/bmj.e1566 22408266

[73] Frankenthal D Israeli A Caraco Y , et al. Long-term outcomes of medication intervention using the screening tool of older persons potentially inappropriate prescriptions screening tool to alert doctors to right treatment criteria. J Am Geriatr Soc 2017; 65: e33–e38. 10.1111/jgs.14570 27943247

[74] Potter K Flicker L Page A , et al. Deprescribing in frail older people: a randomised controlled trial. PLoS One 2016; 11: e0149984. 10.1371/journal.pone.0149984 PMC4778763 26942907

[75] Ruths S Straand J Nygaard HA , et al. Effect of antipsychotic withdrawal on behavior and sleep/wake activity in nursing home residents with dementia: a randomized, placebo-controlled, double-blinded study the Bergen district nursing home study. J Am Geriatr Soc 2004; 52: 1737–1743. 15450054 10.1111/j.1532-5415.2004.52470.x

[76] Bogaerts JM Gussekloo J de Jong-Schmit BE , et al. Effects of the discontinuation of antihypertensive treatment on neuropsychiatric symptoms and quality of life in nursing home residents with dementia (DANTON): a multicentre, open-label, blinded-outcome, randomised controlled trial. Age Ageing 2024; 53: afae133. 10.1093/ageing/afae133 PMC11227112 38970547

[77] Okafor CE Keramat SA Comans T , et al. Cost-consequence analysis of deprescribing to optimize health outcomes for frail older people: A within-trial analysis. J Am Med Dir Assoc 2024; 25: 539–544.e2. 10.1016/j.jamda.2023.12.016 38307120

[78] Ferro-Uriguen A Beobide-Telleria I Gil-Goikouria J , et al. Effectiveness of a person-centered prescription model in hospitalized older people at the end of life according to their disease trajectories and frailty index. Int J Environ Res Public Health 2023; 20: 3542. 36834233 10.3390/ijerph20043542 PMC9967609

[79] M Chess-Williams L M Broadbent A Hattingh L . Cross-sectional study to evaluate patients’ medication management with a new model of care: incorporating a pharmacist into a community specialist palliative care telehealth service. BMC Palliat Care 2024; 23: 172. 39010021 10.1186/s12904-024-01508-1 PMC11251105

[80] Whitty R Porter S Battu K , et al. A pilot study of a medication rationalization (MERA) intervention. CMAJ Open 2018; 6: E87–E94. 10.9778/cmajo.20170134 PMC5878954 29467186

[81] Uchida M Suzuki S Sugawara H , et al. Multicentre prospective observational study on community pharmacist interventions to reduce inappropriate medications. Int J Pharm Pract 2022; 30: 427–433. 35472143 10.1093/ijpp/riac032

[82] Gerardi S Sperlea D Levy SO-L , et al. Implementation of targeted deprescribing of potentially inappropriate medications in patients on hemodialysis. Am J Health Syst Pharm 2022; 79: S128–S135. 10.1093/ajhp/zxac190 35881917

[83] Garfinkel D Zur-Gil S Ben-Israel J. The war against polypharmacy: a new cost-effective geriatric-palliative approach for improving drug therapy in disabled elderly people. Isr Med Assoc J 2007; 9: 430–434. 17642388

[84] Frankenthal D Lerman Y Kalendaryev E , et al. Intervention with the screening tool of older persons potentially inappropriate prescriptions/screening tool to alert doctors to right treatment criteria in elderly residents of a chronic geriatric facility: a randomized clinical trial. J Am Geriatr Soc 2014; 62: 1658–1665. 25243680 10.1111/jgs.12993

[85] Wang Q Zhang J Li K , et al. Effectiveness of different medication management measures in older patients with chronic diseases and polypharmacy: a systematic review and network meta-analysis. Res Soc Adm Pharm 2025; 21: 753–764. 10.1016/j.sapharm.2025.05.011 40451673

[86] Potter K Page A Clifford R , et al. Deprescribing: a guide for medication reviews. J Pharm Pract Res 2016; 46: 358–367.

[87] Brokaar EJ van den Bos F Visser LE , et al. Deprescribing in older adults with cancer and limited life expectancy: an integrative review. Am J Hosp Palliat Med 2022; 39: 86–100. 10.1177/10499091211003078 33739162

[88] Wojtowycz M Morley C Indelicato A , et al. An economic evaluation of prescription drug costs in a deprescribing initiative in a skilled nursing facility system. Econom Policy Anal 2023; 21(Suppl. 3): 5450.

[89] Robinson-Barella A Richardson CL Bayley Z , et al. ‘Do I actually even need all these tablets?’ a qualitative study exploring deprescribing decision-making for people in receipt of palliative care and their family members. Palliat Med 2025; 39: 543–552. 40167136 10.1177/02692163251327900 PMC12033382

