# Supplementary material for: Outcomes of deprescribing for people with life-limiting conditions: A systematic review
Source: Palliat Med. 2026 Feb 22;40(4):490–513. doi: 10.1177/02692163261416281 (PMC13062472; doi:10.1177/02692163261416281)
Supplement: sj-docx-1-pmj-10.1177_02692163261416281 – Supplemental material for Outcomes of deprescribing for people with life-limiting conditions: A systematic review [file sj-docx-1-pmj-10.1177_02692163261416281.docx]

**Supplementary File**

**Title:** Outcome of deprescribing for people with life limiting conditions: A systematic review

​​**Table of Content**

[Supplementary Table 1. PRISMA Checklist  2](#_Toc202526884)

[Search strategy for Different Databases  5](#_Toc202526885)

[Supplementary Table 2. Database: Medline 5](#_Toc202526886)

[Supplementary Table 3. Database: Embase 6](#_Toc202526887)

[Supplementary Table 4. Database: PsycINFO 7](#_Toc202526888)

[Supplementary Table 5. Database: CINAHL 7](#_Toc202526889)

[Supplementary Table 6. Database: SCOPUS 8](#_Toc202526890)

[Supplementary Table 7. Summary of deprescribed medications in people with life limiting conditions 10](#_Toc202526891)

[Supplementary Table 8. Detail of Medication related outcomes reported by Included Studies  13](#_Toc202526892)

[Supplementary Table 9. Detail of Medication related outcomes reported by Included Studies 28](#_Toc202526893)

[Supplementary Table 10. Detail of System outcomes reported by Included Studies 33](#_Toc202526894)

[Critical Appraisal of Included Studies 38](#_Toc202526895)

[Supplementary Table 11. Quality assessment tool for Economic Evaluations 38](#_Toc202526896)

[Supplementary Table 12. Quality assessment tool for Analytical Cross-Sectional Studies 38](#_Toc202526897)

[Supplementary Table 13. Quality assessment tool for Randomised control study 38](#_Toc202526898)

[Supplementary Table 14. Quality assessment of Cohort Studies 39](#_Toc202526899)

[Supplementary Table 15. Quality assessment tool for Quasi-Experimental Studies 40](#_Toc202526900)

# Supplementary Table 1. PRISMA Checklist

| **Section and Topic** | **Item #** | **Checklist item** | **Location where item is reported** |
| --- | --- | --- | --- |
| **TITLE** | | |  |
| Title | 1 | Identify the report as a systematic review. | 1 |
| **ABSTRACT** | | |  |
| Abstract | 2 | See the PRISMA 2020 for Abstracts checklist. | 2 |
| **INTRODUCTION** | | |  |
| Rationale | 3 | Describe the rationale for the review in the context of existing knowledge. | 4-5 |
| Objectives | 4 | Provide an explicit statement of the objective(s) or question(s) the review addresses. | 5 |
| **METHODS** | | |  |
| Eligibility criteria | 5 | Specify the inclusion and exclusion criteria for the review and how studies were grouped for the syntheses. | 6 |
| Information sources | 6 | Specify all databases, registers, websites, organisations, reference lists and other sources searched or consulted to identify studies. Specify the date when each source was last searched or consulted. | 6 |
| Search strategy | 7 | Present the full search strategies for all databases, registers and websites, including any filters and limits used. | 6 |
| Selection process | 8 | Specify the methods used to decide whether a study met the inclusion criteria of the review, including how many reviewers screened each record and each report retrieved, whether they worked independently, and if applicable, details of automation tools used in the process. | 6-7 |
| Data collection process | 9 | Specify the methods used to collect data from reports, including how many reviewers collected data from each report, whether they worked independently, any processes for obtaining or confirming data from study investigators, and if applicable, details of automation tools used in the process. | 6-7 |
| Data items | 10a | List and define all outcomes for which data were sought. Specify whether all results that were compatible with each outcome domain in each study were sought (e.g. for all measures, time points, analyses), and if not, the methods used to decide which results to collect. | 6/Table 1 |
|  | 10b | List and define all other variables for which data were sought (e.g. participant and intervention characteristics, funding sources). Describe any assumptions made about any missing or unclear information. | 6/Table1 |
| Study risk of bias assessment | 11 | Specify the methods used to assess risk of bias in the included studies, including details of the tool(s) used, how many reviewers assessed each study and whether they worked independently, and if applicable, details of automation tools used in the process. | 7 |
| Effect measures | 12 | Specify for each outcome the effect measure(s) (e.g. risk ratio, mean difference) used in the synthesis or presentation of results. | 7-8 |
| Synthesis methods | 13a | Describe the processes used to decide which studies were eligible for each synthesis (e.g. tabulating the study intervention characteristics and comparing against the planned groups for each synthesis (item #5)). | 7-8 |
|  | 13b | Describe any methods required to prepare the data for presentation or synthesis, such as handling of missing summary statistics, or data conversions. | 7-8 |
|  | 13c | Describe any methods used to tabulate or visually display results of individual studies and syntheses. | 7-8 |
|  | 13d | Describe any methods used to synthesize results and provide a rationale for the choice(s). If meta-analysis was performed, describe the model(s), method(s) to identify the presence and extent of statistical heterogeneity, and software package(s) used. | 7-8 |
|  | 13e | Describe any methods used to explore possible causes of heterogeneity among study results (e.g. subgroup analysis, meta-regression). | 7-8 |
|  | 13f | Describe any sensitivity analyses conducted to assess robustness of the synthesized results. | 7-8 |
| Reporting bias assessment | 14 | Describe any methods used to assess risk of bias due to missing results in a synthesis (arising from reporting biases). | 7 |
| Certainty assessment | 15 | Describe any methods used to assess certainty (or confidence) in the body of evidence for an outcome. | Not reported |
| **RESULTS** | | |  |
| Study selection | 16a | Describe the results of the search and selection process, from the number of records identified in the search to the number of studies included in the review, ideally using a flow diagram. | 9/Figure 1 |
|  | 16b | Cite studies that might appear to meet the inclusion criteria, but which were excluded, and explain why they were excluded. | 9/Figure 1 |
| Study characteristics | 17 | Cite each included study and present its characteristics. | 9/Figure 2, 3 |
| Risk of bias in studies | 18 | Present assessments of risk of bias for each included study. | 15 |
| Results of individual studies | 19 | For all outcomes, present, for each study: (a) summary statistics for each group (where appropriate) and (b) an effect estimate and its precision (e.g. confidence/credible interval), ideally using structured tables or plots. | 10-15/Table 2,3 |
| Results of syntheses | 20a | For each synthesis, briefly summarise the characteristics and risk of bias among contributing studies. | 10-15/Table 2,3 |
|  | 20b | Present results of all statistical syntheses conducted. If meta-analysis was done, present for each the summary estimate and its precision (e.g. confidence/credible interval) and measures of statistical heterogeneity. If comparing groups, describe the direction of the effect. | 10-15/Table 2,3 |
|  | 20c | Present results of all investigations of possible causes of heterogeneity among study results. | 10-15/Table 2,3 |
|  | 20d | Present results of all sensitivity analyses conducted to assess the robustness of the synthesized results. | 10-15/Table 2,3 |
| Reporting biases | 21 | Present assessments of risk of bias due to missing results (arising from reporting biases) for each synthesis assessed. | 15 |
| Certainty of evidence | 22 | Present assessments of certainty (or confidence) in the body of evidence for each outcome assessed. | Not reported |
| **DISCUSSION** | | |  |
| Discussion | 23a | Provide a general interpretation of the results in the context of other evidence. | 15-19 |
|  | 23b | Discuss any limitations of the evidence included in the review. | 18-19 |
|  | 23c | Discuss any limitations of the review processes used. | 19-19 |
|  | 23d | Discuss implications of the results for practice, policy, and future research. | 15-19 |
| **OTHER INFORMATION** | | |  |
| Registration and protocol | 24a | Provide registration information for the review, including register name and registration number, or state that the review was not registered. | 5 |
|  | 24b | Indicate where the review protocol can be accessed, or state that a protocol was not prepared. | 5 |
|  | 24c | Describe and explain any amendments to information provided at registration or in the protocol. | 5 |
| Support | 25 | Describe sources of financial or non-financial support for the review, and the role of the funders or sponsors in the review. | 20 |
| Competing interests | 26 | Declare any competing interests of review authors. | 20 |
| Availability of data, code and other materials | 27 | Report which of the following are publicly available and where they can be found: template data collection forms; data extracted from included studies; data used for all analyses; analytic code; any other materials used in the review. | NA |

*From:* Page MJ, McKenzie JE, Bossuyt PM, Boutron I, Hoffmann TC, Mulrow CD, *et al.* The PRISMA 2020 statement: an updated guideline for reporting systematic reviews. BMJ 2021;372:n71. doi: 10.1136/bmj.n71

For more information, visit: <http://www.prisma-statement.org/>

# Search strategy for Different Databases

**Supplementary Table 2. Database: Medline**

| 1 | exp Frailty/ or exp Frail Elderly/ or frail.mp. |
| --- | --- |
| 2 | frailty.mp. or exp Frailty/ |
| 3 | exp Palliative Care/ or exp "Hospice and Palliative Care Nursing"/ or Palliative.mp. |
| 4 | hospice care.mp. or exp Hospice Care/ |
| 5 | exp Terminal Care/ or Terminal care.mp. |
| 6 | ADVANCE MED PLANNING.mp. or exp Advance Care Planning/ |
| 7 | advance care planning.mp. |
| 8 | exp Terminally Ill/ |
| 9 | aged.mp. or exp "Aged, 80 and over"/ or exp Aged/ or elder*.mp. or geriatic.mp. or old people.mp. or older adult.mp. |
| 10 | (end stage or end-stage or terminal stage or end of life or end-of-life or EOL or last year of life or LYOL or life's end).mp. or exp accidental falls/ |
| 11 | (limited life expectancy or life limiting illness or life-limiting illness or limited prognosis or limited expectancy or life limiting condition).mp. |
| 12 | ((Complex or "Longterm" or "long-term" or "long term" or advanced or incurable) and (disease* or ill* or disorder?)).mp. |
| 13 | ((terminal* adj3 (care or caring or patient*)) or progressive illness).mp. |
| 14 | ((advanced or terminal or life threatening) adj5 (ill* or disease*)).mp. |
| 15 | ((limited or diminished or short) adj3 life expectancy).mp. |
| 16 | 1 or 2 or 3 or 4 or 5 or 6 or 7 or 8 or 10 or 11 or 12 or 13 or 14 or 15 |
| 17 | 9 and 16 |
| 18 | 1 or 2 or 3 or 4 or 5 or 6 or 7 or 8 or 10 or 11 or 12 or 13 or 14 or 15 or 17 |
| 19 | (medic* adj5 (withdraw* or discontinu* or ceas* or cessation or deintensif* or de-intensif* or deprescri* or de-prescri* or eliminat* or reduc* or stop* or taper* or unprescri*)).mp. |
| 20 | (polypharmacy or STOPP or STOPPFrail or BEERS or PIM or deprescri* or de-prescrib* or medication review or medic* optimi?ation or Inappropriate Prescri* or Potentially inappropriate medicine or inappropriate medic*).mp. |
| 21 | exp Polypharmacy/ or exp Inappropriate Prescribing/ or exp Potentially Inappropriate Medication List/ or exp deprescription/ |
| 22 | 19 or 20 or 21 |
| 23 | 18 and 22 |
| 24 | limit 23 to (english language and humans and yr="2000-2024") |
| 25 | limit 24 to ("all adult (19 plus years)" or "young adult (19 to 24 years)" or "adult (19 to 44 years)" or "young adult and adult (19-24 and 19-44)" or "middle age (45 to 64 years)" or "middle aged (45 plus years)" or "all aged (65 and over)" or "aged (80 and over)") |
| 26 | limit 25 to (autobiography or case reports or clinical trial, veterinary or clinical trial protocol or editorial or observational study, veterinary or randomized controlled trial, veterinary or "review" or "scientific integrity review" or "systematic review") |
| 27 | 25 not 26 |

**Supplementary Table 3. Database: Embase**

| 1 | exp frail elderly/ or exp Edmonton Frail Scale/ or Frail.mp. |
| --- | --- |
| 2 | exp frailty/ or Frailty.mp. or exp Clinical Frailty Scale/ or exp Tilburg Frailty indicator/ |
| 3 | exp palliative nursing/ or Palliative.mp. or exp palliative therapy/ |
| 4 | hospice.mp. or exp hospice care/ or exp hospice/ or exp hospice nursing/ or exp hospice patient/ |
| 5 | Terminal care.mp. or exp terminal care/ |
| 6 | exp advance care planning/ or Advance care.mp. |
| 7 | Terminally ill.mp. or exp terminally ill patient/ |
| 8 | (end stage or end-stage or terminal stage or end of life or end-of-life or EOL or last year of life or LYOL or life's end).mp. or exp falling/ |
| 9 | (limited life expectancy or life limiting illness or life-limiting illness or limited prognosis or limited expectancy or life limiting condition).mp. |
| 10 | ((Complex or "Longterm" or "long-term" or "long term" or advanced or incurable) and (disease* or ill* or disorder?)).mp. |
| 11 | ((terminal* adj3 (care or caring or patient*)) or progressive illness).mp. |
| 12 | ((advanced or terminal or life threatening) adj5 (ill* or disease*)).mp. |
| 13 | ((limited or diminished or short) adj3 life expectancy).mp. |
| 14 | 1 or 2 or 3 or 4 or 5 or 6 or 7 or 8 or 9 or 10 or 11 or 12 or 13 |
| 15 | exp aged/ or exp home for the aged/ or aged.mp. or exp aged hospital patient/ |
| 16 | geriatric.mp. or geriatrics/ |
| 17 | (elder* or old people or older adult).mp. |
| 18 | 15 or 16 or 17 |
| 19 | 14 and 18 |
| 20 | 14 or 19 |
| 21 | (medic* adj5 (withdraw* or discontinu* or ceas* or cessation or deintensif* or de-intensif* or deprescri* or de-prescri* or eliminat* or reduc* or stop* or taper* or unprescri*)).mp. |
| 22 | exp inappropriate polypharmacy/ or exp polypharmacy/ or Polypharmacy.mp. |
| 23 | exp deprescription/ |
| 24 | exp potentially inappropriate medication/ |
| 25 | (polypharmacy or STOPP or STOPPFrail or BEERS or PIM or deprescri* or de-prescrib* or medication review or medic* optimi?ation or Inappropriate Prescri* or Potentially inappropriate medicine or inappropriate medic*).mp. |
| 26 | 21 or 22 or 23 or 24 or 25 |
| 27 | 20 and 26 |
| 28 | limit 27 to (human and english language) |
| 29 | limit 28 to yr="2000-2024" |
| 30 | limit 29 to ((books or chapter or conference abstract or "conference review" or editorial or letter or note) and (children or children - focussed)) |
| 31 | 29 not 30 |
| 32 | limit 31 to (adult <18 to 64 years> or aged <65+ years>) |
| 33 | limit 32 to embase |

## Supplementary Table 4. Database: PsycINFO

| 1 | exp Geriatrics/ or exp Elder Care/ or exp Geriatric Patients/ or Elder Care/ or older adult.mp. or old people.mp. |
| --- | --- |
| 2 | exp Frail/ or Frailty.mp. or "Frail Elderly".mp. |
| 3 | exp Palliative Care/ or exp Hospice/ or "Hospice Care".mp. or "Palliative Care".mp. or "terminal care".mp. or "Advance Care Planning".mp. or "Advanced practice nursing care".mp. |
| 4 | (End stage or end-stage or terminal stage).mp. or exp "End of Life Care"/ or end-of-life.mp. or end of life.mp. or Accidental Falls.mp. or last year of life.mp. or LYOL.mp. or life's end.mp. |
| 5 | exp Terminally Ill Patients/ or exp Critical Illness/ or exp Chronic Illness/ or (limited life expectancy or life limiting illness or life-limiting illness or limited prognosis or limited expectancy or life limiting condition).mp. |
| 6 | ((complex or "longterm" or "long-term" or "long term" or advanced or incurable) and (disease* or ill* or disorder*)).mp. |
| 7 | ((terminal* adj3 (care or caring or patient*)) or progressive illness).mp. |
| 8 | ((limited or diminished or short) adj3 life expectancy).mp. |
| 9 | 2 or 3 or 4 or 5 or 6 or 7 or 8 |
| 10 | 1 and 9 |
| 11 | 9 or 10 |
| 12 | exp Polypharmacy/ |
| 13 | (polypharmacy or STOPP or STOPPFrail or BEERS or PIM or deprescri* or "de-prescrib*" or "medication review" or "medication optimization" or "medication optimisation" or "inappropriate prescribing" or "potentially inappropriate medicine" or "inappropriate medication").mp. |
| 14 | (medic* adj5 (withdraw* or discontinu* or ceas* or cessation or deintensif* or "de-intensif*" or deprescri* or "de-prescrib*" or eliminat* or reduc* or stop* or taper* or unprescri*)).mp. |
| 15 | 12 or 13 or 14 |
| 16 | 11 and 15 |
| 17 | limit 16 to (human and english language and yr="2000 - 2024") |

**Supplementary Table 5. Database: CINAHL**

| S1 | "frailty" OR (MH "Frailty Syndrome") OR (MH "Frail Elderly") OR "frail" |
| --- | --- |
| S2 | (MH "Palliative Care") OR "palliative care" OR (MH "Palliative Care Nursing") OR (MH "Palliative Medicine") OR "palliative" OR (MH "Hospice Patients") OR (MH "Hospice Care") OR (MH "Terminal Care+") OR "Terminal care" OR (MH "Terminal Care (Saba CCC)+") |
| S3 | (MH "Advance Care Planning") OR "advance care planning" |
| S4 | (MH "Terminally Ill Patients+") |
| S5 | "end stage" OR "end-stage" OR "terminal stage" OR "end of life" OR "end-of-life" OR "EOL" OR "last year of life" OR "LYOL" OR "Life's end" OR (MH "Accidental Falls") |
| S6 | ""((terminal* N3 (care OR caring OR patient*)) OR progressive illness)"" |
| S7 | "( Complex OR "Longterm" OR "long-term" OR "long term" OR advanced OR incurable ) AND ( disease* OR ill* OR disorder* )" |
| S8 | "limited expectancy OR limited prognosis OR limited life expectancy OR life limiting illness OR life-limiting illness OR life limiting condition" |
| S9 | "((advanced OR terminal OR life threatening) N5 (ill* OR disease*))" |
| S10 | "((limited OR diminished OR short) N3 (life expectancy))" |
| S11 | S1 OR S2 OR S3 OR S4 OR S5 OR S6 OR S7 OR S8 OR S9 OR S10 |
| S12 | (MH "Aged+") OR "aged" OR (MH "Aged, 80 and Over+") OR "elder" OR "old people" OR (MH "Older Adult Care (Saba CCC)") OR "older adult" OR "geriatric" |
| S13 | S11 AND S12 |
| S14 | S1 OR S2 OR S3 OR S4 OR S5 OR S6 OR S7 OR S8 OR S9 OR S10 OR S13 |
| S15 | ""(medic* N5 (withdraw* OR discontinu* OR ceas* OR cessation OR deintensif* OR de-intensif* OR deprescri* OR de-prescri* OR eliminat* OR reduc* OR stop* OR taper* OR unprescri*))"" |
| S16 | "(polypharmacy OR STOPP OR STOPPFrail OR BEERS OR PIM OR deprescrib* OR de-prescrib* OR "medication review" OR "medication optimization" OR "medication optimisation" OR "inappropriate prescribing" OR "potentially inappropriate medicine" OR "inappropriate medication")" |
| S17 | (MH "Polypharmacy+") OR (MH "Polypharmacy (Saba CCC)") OR (MH "Inappropriate Prescribing") OR (MH "Deprescribing") |
| S18 | S15 OR S16 OR S17 |
| S19 | (S15 OR S16 OR S17) AND (S14 AND S18) |
| S20 | (S15 OR S16 OR S17) AND (S14 AND S18) |

## Supplementary Table 6. Database: SCOPUS

| 1 | TITLE-ABS-KEY("palliative care" OR "hospice care" OR "terminal care") |
| --- | --- |
| 2 | TITLE-ABS-KEY("terminal stage" OR "end of life" OR "end-of-life" OR "last year of life" OR "terminally ill patients" OR frailty OR "frail elderly" OR frail) |
| 3 | TITLE-ABS-KEY((advanced OR terminal OR "life threatening") W/5 (ill* OR disease*)) |
| 4 | TITLE-ABS-KEY("limited life expectancy" OR "life limiting illness" OR "life-limiting illness" OR "life limiting condition") |
| 5 | TITLE-ABS-KEY(( terminal* W/3 (care OR caring OR patient* )) |
| 6 | ABS("end stage" OR "end-stage" OR "limited expectancy") |
| 7 | TITLE-ABS-KEY((Complex OR advanced OR terminal OR "life threatening") W/5 (ill* OR disease*)) |
| 8 | 1 OR 2 OR 3 OR 4 OR 5 OR 6 OR 7 |
| 9 | TITLE-ABS-KEY(elder* OR "old people" OR "older adult" OR geriatric) |
| 10 | 8 AND 9 |
| 11 | 8 OR 10 |
| 12 | Title-abs-key(polypharmacy OR stopp OR stoppfrail OR beers OR pim OR deprescrib* OR "de-prescrib*" OR "medication review" OR "medication optimization" OR "medication optimisation" OR "inappropriate prescribing" OR "potentially inappropriate medicine" OR "inappropriate medication") |
| 13 | TITLE-ABS-KEY((medic*) W/5 ( withdraw* OR discontinu* OR ceas* OR cessation OR deintensif* OR "de-intensif*" OR deprescri* OR "de-prescrib*" OR eliminat* OR reduc* OR stop* OR taper* OR unprescri*)) |
| 14 | 12 OR 13 |
| 15 | 11 AND 14 |
| 16 | PUBYEAR > 1999 AND PUBYEAR < 2025 AND ( LIMIT-TO ( LANGUAGE,"English" ) ) AND ( LIMIT-TO ( EXACTKEYWORD,"Human" ) OR LIMIT-TO ( EXACTKEYWORD,"Humans" ) ) AND ( LIMIT-TO ( DOCTYPE,"ar" ) ) |

# Supplementary Table 7. Summary of deprescribed medications in people with life limiting conditions

| **Included Studies (Author, year)** | **A1** | **A2** | **A3** | **B1** | **B2** | **B3** | **C1** | **D1** | **D2** | **D3** | **D4** | **E1** | **F1** | **F2** | **F3** | **F4** | **F5** | **G1** | **G2** | **H1** | **I1** | **I2** | **Others** |
| --- | --- | --- | --- | --- | --- | --- | --- | --- | --- | --- | --- | --- | --- | --- | --- | --- | --- | --- | --- | --- | --- | --- | --- |
| Basri *et al. ^1^*, 2018 |  | Y |  |  |  |  |  |  |  |  |  |  |  |  |  |  |  |  |  |  |  |  |  |
| Hurley *et al.^2^*, 2024a | Y | Y | Y | Y | Y |  |  | Y |  | Y |  | Y | Y | Y | Y | Y |  |  | Y | Y | Y | Y |  |
| Curtin *et al.*^3^,2020 * | Y | Y |  | Y | Y |  | Y | Y |  | Y |  | Y | Y |  | Y |  |  |  |  | Y | Y | Y | ^a^ |
| Niznik *et al.^4^*, 2022 |  |  |  |  |  |  |  |  |  |  |  |  |  |  |  |  |  |  |  | Y |  |  |  |
| Niznik *et al.^5^*, 2020a |  |  |  |  |  |  |  |  |  | Y |  |  |  |  |  |  |  |  |  |  |  |  |  |
| Brunet *et al.*, 2014 |  |  |  | Y | Y |  | Y |  | Y | Y | Y | Y |  |  | Y | Y |  |  | Y | Y |  | Y | ^b^ |
| Hurley *et al.^6^*, 2024b | Y | Y | Y | Y | Y |  |  | Y |  | Y |  | Y | Y | Y | Y | Y |  |  | Y | Y | Y | Y |  |
| Niznik *et al.^7^*, 2020b |  |  |  |  |  |  |  |  |  | Y |  |  |  |  |  |  |  |  |  |  |  |  |  |
| Kutner *et al.^8^,* 2015 |  |  |  | Y |  |  |  |  |  |  |  |  |  |  |  |  |  |  |  |  |  |  |  |
| Tse *et al. ^9^*, 2008 |  |  |  |  |  |  |  |  |  |  |  |  |  |  |  |  |  |  |  |  |  |  | ^c^ |
| M Chess-Williams *et al. ^10^*, 2024 |  | Y |  | Y | Y |  | Y |  |  |  |  | Y | Y |  |  | Y | Y |  |  | Y | Y | Y |  |
| Whitman *et al. ^11^*, 2018 |  |  |  | Y | Y |  | Y |  | Y |  |  | Y |  |  |  | Y |  |  |  |  | Y | Y |  |
| Bergh *et al. ^12^*, 2012 |  |  |  |  |  |  |  |  |  |  | Y |  |  |  |  |  |  |  |  |  |  |  |  |
| Saad *et al. ^13^*, 2012 |  |  |  | Y | Y |  |  |  | Y |  | Y | Y |  |  |  | Y |  |  |  | Y |  | Y | ^d^ |
| Frankenthal *et al. ^14^*, 2017 |  |  |  |  | Y |  | Y | Y | Y | Y |  | Y |  |  |  | Y | Y | Y | Y | Y |  |  | e |
| Potter *et al. ^15^*, 2016 |  |  |  | Y | Y | Y | Y | Y | Y | Y | Y | Y | Y | Y | Y | Y | Y |  |  | Y | Y | Y | ^f^ |
| Etherton-Beer *et al. ^16^*, 2023 |  |  |  | Y | Y | Y | Y | Y | Y | Y | Y | Y | Y | Y | Y | Y | Y | Y | Y | Y | Y | Y | ^g^ |
| Poudel *et al. ^17^*, 2015 |  |  |  |  | Y |  |  | Y | Y |  | Y |  |  |  |  | Y |  |  |  |  |  |  | ^h^ |
| Brunetti *et al. ^18^*, 2024 |  |  |  |  |  |  | Y |  |  |  |  |  |  |  |  |  |  |  |  |  |  |  |  |
| Nakagaito *et al. ^19^*, 2024 |  |  |  |  |  |  |  |  |  |  |  |  |  |  |  |  |  |  |  | Y |  |  |  |
| Caravaca *et al. ^20^*, 2018 |  |  |  |  |  |  |  |  |  |  |  |  |  | Y |  |  |  |  |  |  |  |  |  |
| Tapper *et al. ^21^*, 2022 |  |  |  |  |  |  |  |  | Y |  |  |  |  |  |  |  |  |  |  |  |  |  |  |
| Ruths *et al. ^22^*, 2004 |  |  |  |  |  |  |  | Y |  |  |  |  |  |  |  |  |  |  |  |  |  |  |  |
| Bogaerts *et al. ^23^*, 2024 |  |  |  |  | Y |  |  |  |  |  |  |  |  |  |  |  |  |  |  |  |  |  |  |
| Gerardi *et al. ^24^*, 2022 |  |  |  | Y |  | Y | Y |  | Y |  |  | Y |  |  |  |  |  | Y |  |  |  |  | ^i^ |
| Garfinkel *et al. ^25^*, 2007 |  |  |  |  | Y | Y |  | Y | Y |  | Y | Y |  |  |  |  |  |  |  |  |  | Y | ^j^ |
| Malik *et al. ^26^*, 2019 |  |  |  |  |  |  |  |  |  |  |  |  |  |  |  |  |  |  |  |  |  |  | ^k^ |
| Daiello *et al. ^27^*, 2009 |  |  |  |  |  |  |  |  |  | Y |  |  |  |  |  |  |  |  |  |  |  |  |  |
| Wauters *et al. ^28^,* 2021* |  |  |  |  |  |  |  |  |  |  |  | Y |  |  |  |  |  |  | Y |  |  |  |  |
| Okafor *et al. ^29^*, 2024 |  |  |  | Y | Y | Y | Y |  | Y |  |  | Y | Y | Y |  | Y |  |  |  | Y |  |  |  |
| Suhrie *et al. ^30^*, 2009 |  |  |  |  |  |  | Y | Y |  |  |  |  |  |  |  |  |  |  |  | Y | Y |  | ^l^ |
| Yeh *et al. ^31^*, 2013 |  |  |  |  |  |  |  |  |  |  |  |  |  |  |  |  |  |  | Y |  |  |  |  |
| Hayes *et al. ^32^*, 2023 |  |  |  |  |  |  | Y |  |  |  |  |  |  |  |  |  |  |  |  |  |  |  |  |
| Czikk *et al. ^33^*, 2022 |  |  |  |  |  |  |  |  |  |  |  | Y |  |  |  |  |  |  |  |  |  |  |  |
| Whitty *et al. ^34^*, 2018* |  |  |  | Y | Y | Y | Y |  | Y |  |  | Y |  |  | Y | Y |  |  |  |  | Y | Y | ^m^ |
| Riveras *et al. ^35^*, 2024 |  |  |  |  |  |  | Y |  |  |  |  |  |  |  |  |  |  |  |  |  |  |  |  |
| Chin-Yee *et al. ^36^*, 2022 |  |  |  |  |  |  | Y |  |  |  |  |  |  |  |  |  |  |  |  |  |  |  |  |
| Bravo-Jose *et al. ^37^*, 2019 |  |  |  |  |  |  |  | Y |  |  |  |  |  |  |  |  |  |  |  |  |  |  |  |
| Choukroun *et al. ^38^*, 2021* |  |  |  |  |  |  |  |  |  |  |  | Y |  |  |  | Y |  |  |  |  |  |  | ^n^ |
| Kearney *et al.*, 2023 |  |  |  |  | Y |  |  |  |  |  |  |  |  |  |  |  |  |  |  | Y | Y |  |  |
| Suzuki *et al. ^39^*, 2023 |  |  |  | Y | Y | Y | Y | Y | Y | Y | Y | Y | Y |  |  | Y | Y |  |  | Y | Y |  | ^o^ |
| Shirley *et al. ^40^*, 2021 |  | Y |  | Y |  |  | Y |  |  |  |  | Y |  |  |  | Y |  | Y | Y | Y | Y | Y | ^p^ |
| Pruskowski *et al. ^41^*, 2017 |  |  |  |  | Y |  |  |  |  |  |  | Y |  | Y |  |  |  |  |  |  | Y |  |  |
| McIntyre *et al. ^42^*, 2017 |  |  |  | Y |  | Y |  |  |  |  |  | Y |  |  |  |  |  |  |  |  |  |  | ^q^ |

A1: Any drug that the patient persistently fails to take or tolerate despite adequate education and consideration of all appropriate formulations, A2: Any drug without a clear or no clinical indication, A3: Any drug for symptoms which have now resolved (e.g. pain, nausea, vertigo, pruritus), B1. Lipid lowering therapies, B2: Antihypertensive therapies, B3: Diuretics, C1: Anti-thrombotic, D1: Antipsychotics, D2: Benzodiazepine, D3: Antidementia (including Memantine and cholinesterase inhibitors), D4: Antidepressant, E1: Acid Suppressant (Proton Pump Inhibitors and H2 receptor antagonist), F1.: Calcium supplementation, F2: Vitamin D, F3: Anti-resorptive/bone anabolic drugs, F4: Analgesics (including NSAIDs and Opioid), F5: Steroids, G1: Drugs for benign prostatic hyperplasia (including mirabegron), G2: Anticholinergic, H1: Diabetic drugs, I1: Vitamin supplements, I2: Nutritional supplements; *indicates medications targeted for deprescribing,

^a^Leukotrine antagonist and Alpha blockers with long-term bladder catheterisation; ^b^Theophylline, antispasmodics, eyedrops, low value intrinsic medications, brain vasodilators and nootropics drugs, chondroprotectors; ^c^Levodopa; ^d^Bowel regiment agents, thyroid, antiarrhythmic; ^e^duplicated prescriptions, anti-diarrhoeal, H1 anti-histamine, digoxin, theophylline; ^f^Laxatives, antifungal, antibacterial, antigout, antirheumatic, anti-parkinsonism, anti-epileptic, drugs for chronic obstructive pulmonary disease and ophthalmology; ^g^H1 Anti-histamine, drugs for obstructive airway, anti-vertigo, antiepileptics, anti-pyretics, drugs affecting bone structure and mineralisation, anti-gout, muscle relaxants, anti-inflammatory, anti-rheumatic, antibacterial, lactobacillus, anti-haemorrhoids vasodilators, digoxin, ubidecarenone, digestives, drugs laxatives, anti-emetics; ^h^anti-arrhythmic, antispasmodic; ^i^Allopurinol; ^j^Pentoxifylline, anti-anginal; ^k^Digoxin; ^l^Gastrointestinal agents (e.g. docusate); ^m^Nonbenzodiazepine hypnotics and docusate; ^n^hypnotics and anxiolytics; ^o^antiflatulants, mucosal protection agents, anti-epileptic, non-benzodiazepine hypnotics, antiemetics, laxatives, herbal medicines, antibacterials, airway mucus regulators, antiallergic, anti-cancer, anti-arrhythmic, antitussives, hepatobiliary protection agents, rehydration agents, circulation improvers, k-replacement medications, muscle relaxants, ^p^cardiac medication (excluding statins or aspirin), wound/skin care agents, gastrointestinal or bowel medications (excluding PPIs or docusate), mouth/throat care, docusate, memory agents, pulmonary medications, allopurinol, hydroxyzine, lamotrigine, antibiotics, hypnotics, ^q^Quinine and alpha1 blocker,

# Supplementary Table 8. Detail of Medication related outcomes reported by Included Studies

| **Identity** | **Reported time (months)** | **Cognitive and Neuropsychiatric Outcomes** | **Functional and Physical health outcome** | **Behavioural and Mood Outcome** | **Mortality and survival outcome** | **Quality of life outcome** | **Clinical events and complication outcome** |
| --- | --- | --- | --- | --- | --- | --- | --- |
| Hurley *et al.^6^*, 2024b | 6 months | **Anticholinergic Cognitive Burden** (mean, SD): BI: 4.27, 2.45, AI at 6 months: 3.94, 2.70, p = **.032** | **Number of patients with Falls (**no of events)**:** BI: 21 (36), AI: 17 (31), 13.9% reduction |  |  | **EQ-5D-5L Summary Score** (mean, SD)**:**  BI: 0.183 (0.286), AI: 0.159 (0.312), p = 0.18  **EQ-5D-5L VAS Score** (mean, SD):  BI: 60.3 (22.8), AI: 61.8 (19.9), p = 0.45 |  |
| Daiello *et al. ^27^*, 2009 | Monthly change (Specific time not clear) | **Change in Cognitive performance Scale:** IG: 0.06 Vs CG: 0.02, p =0.38  **(**-ve difference indicate improvement**)** | **Change in ADL Hierarchy Scale:** IG: 0.10 Vs CG: 0.03, p = 0.10  **Change in time spent in leisure-related activities**: IG 0.11, CG: -0.26, p =**0.01**  **(**-ve difference indicate improvement**)** | **Behavioural Symptoms:**  Aggressive Behaviour scale: IG: 0.08, CG: -0.01, p=**0.03**  Socially inappropriate or disruptive behaviour: IG: 0.10, CG: -0.04, p=**0.02**  **Mood Symptoms:** Depression Rating Scale: IG: -0.01, CG: -0.05, p=0.28  Repetitive questioning: IG:0.09, CG: -0.08, p=**0.01**  Repetitive health complaints: IG:0.09, CG: -0.07, p=**0.01**  **(**-ve difference indicate improvement**)** |  | **Change in Bowel Continence**: IG:0.04, CG: 0.04, p=0.98  **Change in bladder continence**: IG: 0.05 Vs CG:0.04, p=0.80 |  |
| Tapper *et al. ^21^*, 2022 | 36 months |  | **Falls, %:** IG-Zolpidem: sHR (95% CI) = 0.84 (0.70-0.99), p = **0.04**  IG-Benzodiazepam: sHR (95% CI) = 0.96 (0.79-1.16), P =0.7  **Fracture, %:** IG-Zolpidem: sHR (95% CI) = 0.80 (0.66-0.97), P = **0.02**  IG-Benzodiazepam: sHR (95% CI) = 0.98 (0.80-1.20), P =0.8 |  | **Mortality, %**: IG-Zolpidem: 29.1%, CG: 33.2%, p = 0.2  IG-Benzodiazepam: 23.3%, CG: 22.9%, P =1  **Time to death, years (IQR):** IG-Zolpidem: 2.0 (0.7-3.7), CG: 2.7 (1.2-4.8)  IG-Benzodiazepine: 1.6 (0.6-3.1), CG: 1.6 (0.7-3.1) |  | **Hepatic Encephalopathy, %:** IG-Zolpidem: sHR (95% CI) = 1.03 (0.75-1.41), p = 0.9  IG-Benzodiazepam: sHR (95% CI) = 0.96 (0.69-1.35), p =0.8  **Ascites, %:** IG-Zolpidem: sHR (95% CI) = 0.87 (0.69-1.08), p = 0.2  IG-Benzodiazepam: sHR (95% CI) = 1.02 (0.80-1.30), p =0.9  **Intracranial Haemorrhage, %:** IG-Zolpidem: sHR (95% CI) = 0.65 (0.35-1.23), p = 0.2  IG-Benzodiazepam: sHR (95% CI) = 0.90 (0.47-1.14), p =0.7 |
| Niznik *et al.^7^*, 2020b | variable |  | **Fall or Fracture in 8.5 months (**events per person-year**):** aOR (95% CI) = 0.64(0.56-0.73), p= **<0.001** |  |  |  |  |
| Kutner *et al.^8^*, 2015 | variable |  | **Australia-Modified Karnofsky Performance Status, mean score at 12 months:** IG: 47.7, CG: 48.5, p-Value:0.63 |  | **Number of deaths at 2 months:** IG: 45 (23.8%), C: 39(20.3%), p=0.36 | **Edmonton Symptom Assessment System scores in mean at 6 months:** IG: 25.1, CG=27.5, p = 0.14  **McGill Qol at 6 months:** IG: 7.07, CG: 6.74, p=**0.03** (higher in discontinuation group) | **Number of cardiovascular events**: IG: 13, CG: 11 |
| Ruths *et al. ^22^*, 2004 | 1 months | **Neuropsychiatric Inventory Questionnaire (NPI-Q), score:** F=2.91, p =0.06 |  | **Sleep efficiency:** F=3.8, P=**0.03** (decreased in IG),  **Total 24-hour activity**: F=0.73, p = 0.49  **Day activity:** F=0.83, p=0.44  **Night activity**: F=0.75, P=0.47 |  |  |  |
| Bergh *et al. ^12^*, 2012 | 25 weeks | **Neuropsychiatric Inventory-10,** mean (SD)**:** IG: 22.54(18.58), CG:14.74(9.15), p=0.056  **Severe impairment battery,** mean (SD)**:** IG: 73.35(23.96), CG:67.97(30.91), p=0.956  **Clinical dementia rating:** No significant difference in all scores (score-1,2,3) (p=>0.05) | **Lawton and Brody’s physical self-maintenance scale,** mean (SD)**:** IG: 18.46 (5.59), CG:18.11 (5.38), p=0.915  **Unified Parkinson’s disease rating scale** (mean, SD)**:** IG: 3.63,3.32, CG: 3.76,3.89, p=0.377 | **Cornell scale of depression in dementia,** mean (SD)**:** IG: 6.03(4.76) CG:4.42(3.77) p=**0.045** |  | **Quality of life-Alzheimer’s disease scale, patients’ rating,** mean (SD)**:** IG:32.80 (7.12) CG: 35.87 (4.95), p=0.314  **Quality of life-Alzheimer’s disease scale, caregivers’ rating,** mean (SD)**:** IG:29.89 (6.57), CG: 29.11 (5.12), p=0.674 | **#Number of Psychotropic drugs, mean (SD):** IG: 1.77 (0.97), CG: 1.87, (1.00), p=0.170 |
| Tse *et al. ^9^*, 2008 | 1 month | **MMSE, mean (SD): I**G: 6.60 (9.84), CG: 9.80 (8.76), No statistical difference  **Nursing Assistant Behavioural Detection Form, mean (SD):** IG:28% (14%), CG: 23% (16%), No statistical difference | **United Parkinson’s Disease Rating Scale, mean (SD):** IG: 41.21 (24.22), CG: 53.20 (16.12), No statistical difference  **Motor and behavioural deterioration**:  Motor deterioration: IG: 0% in I, CG: 0%  Hallucination: IG: 0%, CG: 8%  Confusion: IG: 44%, CG: 16%,  Agitation: IG: 0%, CG: 12%  Psychosis: IG:0%, CG: 0%,  No statistical difference |  |  |  |  |
| Curtin *et al.*^3^, 2020 | 3 months |  | **Fall, proportion (95%CI):** IG: 14(0.17, 0.40), CG: 14 (0.19, 0.44, RR (95% CI): 0.90 (0.48, 1.69), p=0.75  **Non-vertebral Fractures, proportion (95%CI):** IG: 1 (0, 0.11), CG: 4 (0.03, 0.20), RR (95% CI): 0.23 (0.03, 1.95), p=0.18 |  | **Mortality, proportion (95%CI)**: IG: 12/65 (0.11,0.3) CG:18/65 (0.18, 0.4), RR (95% CI): 0.67 (0.35, 1.27), p=0.22 | **Change in ICECAP-O score, mean (SD):** IG: -0.39 (0.36), CG: -0.30 (0.35), p = 0.17,  **Change in QUALIDEM score, mean (SD):** IG: -2.43 (4.65), CG: -2.85 (4.64); p = 0.60 |  |
| Malik *et al. ^26^*, 2019 | 48 months |  |  |  | **All-cause Mortality:** HR: 1.12; 95% CI, 0.98–1.28, p=0.098 |  |  |
| Frankenthal *et al. ^14,43^*, 2017 | 12 months |  | **Change in Functional Independence Measure, mean:** IG: 4.1, CG: 3.5 p=0.14  **Changes in number of falls at 24 months, mean difference:** IG: 0.47, CG: 0.7, p = 0.4) |  |  | **Quality of life using Medical Outcomes Study 12-item Short-Form Health Survey (SF-12)**:  Change in Physical Component: IG: -1.1, CG: 0.4, p = 0.09  Change in Mental Component: IG: 0.7, CG: 0.2, p = 0.70 |  |
| Garfinkel *et al. ^25^*, 2007 | 12 months |  |  |  | **Mortality rate, %**: IG: 21%, CG: 45%, p= <**0.001** |  |  |
| Brunetti *et al. ^18^*, 2024 | 12 months |  |  |  | **All-cause mortality, %**: IG: 56.6%, CG: 37.6%, p = **<0.001**  **Survival time, median days (IQR):** IG: 34 (11-140), CG: 107 (35-231) |  | **Stroke or Systemic embolism, n (%):** IG: 5(1.5%), CG: 36 (2.9), p =0.14  **Major or clinically relevant nonmajor bleeding, n (%):** IG: 14 (4.1%), CG: 60 (4.9), p=0.57 |
| Etherton-Beer *et al. ^16^*, 2023 | 12 months | **Change in MMSE, mean**±SD**:** IG-Open: 14.8 ± 10.5, CG: 14.2 ± 10.5, p = 0.75  IG-Blind: 9.8 ± 10.2, CG: 14.2 ± 10.5, p = **0.01**  **10-Item Neuro-Psychiatric Inventory (NPI),** **mean**±SD**:** IG-Open: 13.9 ± 16.8, CG: 13.1 ± 16.8, p = 0.54  IG-Blind: 15.1 ± 15.9, CG: 13.1 ± 16.8, p = 0.47  **12-Item NPI,** **mean**±SD**:** IG-Open: 16.1 ± 20.5, CG: 15.3 ± 18.4, p = 0.82  IG-Blind: 17.1 ± 17.3, CG: 15.3 ± 18.4, p = 0.54  **10-Item NPI Distress,** **mean**±SD**:** IG-Open: 5.1 ± 5.8, IC: 4.9 ± 5.9, p = 0.80  IG-Blind: 5.8 ± 6.2, CG: 4.9 ± 5.9, p = 0.34  **12-Item NPI Distress,** **mean**±SD**:** IG-Open: 5.9 ± 7.2, CG: 5.6 ± 6.6, p = 0.83  IG-Blind: 6.6 ± 6.8, CG: 5.6 ± 6.6, p = 0.36 | **Independence in activities of daily living by MBI,** **mean**±SD**:** IG-Open: 47 ± 34, IC: 43 ± 33, p = 0.49  IG-Blind: 34 ± 33, IC: 43 ± 33, p = 0.12  **Frailty,** **mean**±SD**:** IG-Open: 0.33 ± 0.07, CG: 0.32 ± 0.10, p = 0.74  IG-Blind: 0.33 ± 0.10, CG: 0.32 ± 0.10, p = 0.52  **Falls per resident, median (IQR):** IG-Open: 0(2), CG: 0(2),  IG-Blind: 0(2), CG: 0(2)  **Fractures per resident, (n, %):** IG-Open: 9(9%), CG: 12(12%),  IG-Blind: 6(6%), CG: 12(12%) |  | **Mortality, n (%):** IG-Open: 28(28%), CG: 20(20%), HR 1.47, 95% CI 0.83, 2.61, p = 0.19  IG-Blind: 20(20%), CG: 20(20%), HR 0.93, 95% CI 0.50, 1.73, p = 0.83 | **Quality of Life EQ-5D-5L,** **mean**±SD**:** IG-Open: 0.61 ± 0.29, CG: 0.58 ± 0.29, p = 0.57  IG-Blind: 0.51 ± 0.32, CG: 0.58 ± 0.29, p = 0.167 |  |
| Potter *et al. ^15^*, 2016 | 12 months | **Change in MMSE, mean (SD):** IG: -3 (5), CG: -2 (4), p=0.60  **Change in NPI-NH, mean (SD):** IG: -0.1(4.7), CG: -0.2(2.3), p=0.95 | **MBI, mean (SD):** IG: -10(17), CG: -11(15), p=0.76  **Falls, proportion (95% CI):** IG: 0.56 (0.42, 0.69), CG: 0.65 (0.50, 0.77), p=0.40  **Fractures, proportion (95% CI):** IG: 0.07 (0.02, 0.19), CG: 0.04 (0.004, 0.15), p=0.67 | **PSQI, mean (SD)**: IG: 0 (3), CG: -1 (2), p=0.76 | **Mortality, n(%):** IG: 12(26%), CG: 19(40%), HR (95% CI): 0.60 (0.30,1.22, p=0.16 | **EQ-5D, mean (SD)**: IG: -11 (17), CG:7 (15), p=0.35  **QOLAD, mean (SD):** IG: -1 (4.3), CG: -1(4.7), p=0.91 | **Change in bowel function:** No significant difference (p=>0.05) in bowel motions and faecal incontinence |
| Bogaerts *et al. ^23^*, 2024 | 8 months | **Change in NPI-NH^b^**: adjusted mean difference ^a^ (95% CI) = 6.2 (1.9 to 10.6), p = **0.0050**  **Change in NPI-NH Caregiver distress ^b^**: adjusted mean difference ^a^ (95% CI) = 2.7 (0.8 to 4.7), p = **0.0060**  **MDS-CPS^d^**: aOR = 0.84 (95% CI: 0.43 to 1.66), p=0.84  **Short CAM^e^**: aOR = 3.07 (95% CI: 1.23 to 7.66), p=**0.017**  **AES-10 ^b^**: IG: 1.6 (6.9), CG: 2.0 (6.9), p = 0.51 | **Change in CDS^c^**: adjusted mean difference (95% CI) = -1.4 (-5.1 to 2.3), p=0.47  **Falls^f^:** aMR = 2.21 (95% CI: 1.56 to 3.13), p=**<0.001**  **Change in Katz-15^b^**: IG: 1.0 (2.4), CG: 0.6 (1.4), p=0.41 |  | **Mortality, n (%)**: IG: 33, (32.7%), CG: 23 (22.1%), adjusted HR (95% CI) 1.65(0.95–2.85), p = 0.074  **Time to death in days, median (IQR)**: IG: 134(68-208), CG: 111(56-173) | **Change in Qualidem^c^**: adjusted mean difference (95% CI) = -3.5 (-8.1 to 1.1), p = 0.13 **Change in CarerQoL-7D index^c^**: adjusted mean difference (95% CI) = 0.4 (-3.9 to 4.8), p=0.84  **Change in DS-DAT^b^**: adjusted mean difference (95% CI) = 0.7 (0.0 to 1.3), p=**0.046**  **Change in CarerQoL-7D- VAS^c^**: adjusted mean difference (95% CI) = -0.4 (-0.9 to 0.1), p=0.11 | **Change in systolic blood pressure ^b^:** adjusted mean difference (95% CI): 4.9 (-0.8 to 10.6), p = 0.089  **Change in diastolic blood pressure ^b^:** adjusted mean difference (95% CI): 3.3 (-0.5 to 7.2), p = 0.088  **#Change in Psychotropic medication use, mean (SD)**: adjusted mean difference (95% CI) = 0.05(-0.10 to 0.21), p=0.49 |
| Niznik *et al.^5^*, 2020a | 9 months |  |  | **Effects on Aggressive Behaviour Scale:** aOR (95% CI) = 0.002 (-0.036,0.041), p = 0.901 |  |  | **#Need for antipsychotic prescribing:** IG: 2%, CG: 5.8%, aOR (95% CI) = 0.52(0.40, 0.68), p=**<0.001** |
| Caravaca *et al. ^20^*, 2018 | 11 months (318 days) |  |  |  | **Mortality, n (%)**: IG: 1(0.01%), CG: 4(0.06) |  | **Reduction in GFR, median (IQR):** IG: -0.17(-0.37; -0.01), CG: -0.30 (-0.56, -0.07), p = 0.059  **Need for Dialysis, n (%)**: IG: 31(46), CG: 29(43) |
| Niznik *et al.^4^*, 2022 | 2 months |  |  |  | **Mortality, proportion (95% CI):** IG: 0.076(0.051,0.103), CG: 0.050(0.029,0.074), Risk Difference: 0.026 (-0.007, 0.061) and Risk Ratio: 1.52 (0.89, 2.81) |  |  |
| Yeh *et al. ^31^*, 2013 | 3 months | **Change in MMSE, mean ± SD:** IG: -0.8± 2.2, CC: -0.4 ± 1.9, p = 0.734  **Change in Clinician-Rate Anticholinergic Score, mean ± SD:** IG: -0.5± 1.1, CC: 0.1 ± 0.5, p = **0.014** | **Change in Barthel Index, mean ± SD:** IG: -0.5± 11.3, CC: -4.3 ± 11.8, p = 0.116 |  |  |  |  |
| Czikk *et al. ^33^*, 2022 | variable |  |  |  | 1 out of 29 died after 2 days of PPIs switched to every second day dosing |  | **Change in serum mineral in 8 to 12 weeks, mean (SD)**  **On Calcium,** mmol/L**:** BI: 2.34(0.12), AI: 2.31(0.18), p = 0.17  **On Phosphate,** mmol/L**:** BI: 1.55(0.29), AI: 1.85(0.34), p = **0.005**  **On Magnesium,** mmol/L**:** BI: 1.01(0.16), AI: 1.06(0.14), p = **0.01** |
| Hayes *et al. ^32^*, 2023 | variable |  |  |  | **All-cause mortality at 0.67 mean years follows up,** deaths per 100 person-years**:** I: 45.6, C: 45.1, HR (95% CI) = 0.99 (0.92 to 1.06) |  | **Major bleeding events at 0.65 mean years follows up,** events per 100 person-years**:** I: 8, C: 9.4, HR (95% CI) = 1.18 (1.03 to 1.37) **Thrombotic events**^g^ **at 0.67 mean years follows up,** events per 100 person-years**:** I: 5.5, C: 6.4, HR (95% CI) = 1.16 (0.96 to 1.41) |
| Riveras *et al. ^35^*, 2024 | variable |  |  |  |  |  | **Major bleeding or VTE, n (%):** IG: 7 (9.9%) on 56 median days, CG: 4 (13.8%) on 31 median days, p = 0.726 |
| Chin-Yee *et al. ^36^*, 2022 | 111 median days |  |  |  | **All-cause mortality, per 100 person-years (95% CI):** IG: 135.9(129.8-142.3), CG: 95.7(93.2-98.3), HR = 1.35 (1.28-1.42) |  | **Thrombotic event‡, per 100 person-years (95% CI):** IG: 5.2 (4.1-6.6), CG: 4.9(4.3-5.5), HR = 1.06 (0.81-1.39)  **Bleeding event§, per 100 person-years (95% CI):** IG: 10.4(8.7-12.3), CG: 12.7(11.8-13.7), HR = 0.75 (0.62-0.90) |
| Bravo-Jose *et al. ^37^*, 2019 | 6 months | **NPI-NH, mean** + **SD:** BI: 12.91+12.80, AI: 13.76+ 16.68, p = 0.125 |  |  |  |  |  |
| Kearney *et al.^44^*, 2023 | 12 months |  |  |  | **Mortality (%):** IG: 33%, CG: 67%, p = **0.02**  **Time (months) to death, median (IQR):** IG: 4.9(1.9-8.1), CG: 4.5 (2.5-9.6), p = 0.57 |  |  |
| Ruderman *et al. ^45^*, 2018 | 12 months |  | **Fracture patients, number**: IG: 2, CG: 5, p = >0.05 |  | **Mortality, number:** IG: 13, CG: 10, p = >0.05 |  | **Biochemical changes in Serum:**  **PTH level (pmol/L), median (IQR):** IG: 114.8(83.9-159.1), CG: 41.3(28.5-69.7), p = **<0.005**  **Calcium level (mmol/L), mean (SD):** IG: 2.46(0.14), CG: 2.32(0.14), p =**<0.005**  **Phosphate level (mmol/L), mean (SD):** IG: 1.77(0.58), CG: 1.6(0.49), p = 0.14  **Alkaline Phosphatase level (IU/L), mean (SD)**: IG: 161.7(72.9), CG: 121.5(45), p = **0.003**  **Albumin level (g/L), mean (SD):** IG: 33(4.5), CG:34.3(5), p=0.94  **CRP level (mg/L), median (IQR):** IG: 7.7(3-29.5), CG: 6.3(2-19), p=0.41  **Ferritin level (microg/L), median (IQR):** IG: 274.5(120-383.5), CG: 221.5(138.5-322.8), p=0.64  **Haemoglobin level (g/L), mean (SD):** IG: 109(14), CG: 114(14), p=0.09  **Bicarbonate level (g/L), mean (SD):** IG: 23.6(3.2), CG: 24(3), p=0.81  **25-hroxy vitamin D level (nmol/L), median (IQR):** IG: 53(28-70), CG: 60(42-97), p = 0.36  **Parathyroidectomy and referral for surgery, number:** IG: 5, CG: 1, p = >0.05,  **Calciphylaxis, number**: IG: 1, CG: 0, p =>0.05 |

BI: Before Intervention, AI: After Intervention, SD: Standard deviation, IG: Intervention group, CG: Control or comparator group, IQR: Interquartile range, EQ-5D-5L: EuroQol 5-Dimension 5-Level Questionnaires, EQ-5D-5L VAS score: EuroQol 5-Dimension 5-Level Visual Analogue Scale (VAS), ADL: Activities of Daily Living, sHR: Subdistribution Hazardous Ratio, MMSE: Mini-Mental State Examination**,** aOR: adjusted Odd Ratio, aMR: adjust Mean Ration, RR: Relative Risk, HR: Hazardous Ratio, MBI: Modified Barthel Index, NPI-NH: Neuropsychiatric Index – Nursing Home Version, QOLAD: Quality of Life in Alzheimer’s Dementia; PSQI: Pittsburgh Sleep Quality Index, MDS-CPS: Minimum Data Set Cognitive Performance Scale, CAM: Confusion Assessment Method, AES-10: Apathy Evaluation Scale-10, CDS: Care Dependency Scale, Qualidem: indicates Dementia-specific quality of life assessment scale, CarerQoL-7D: Care-related quality-of-life-7 Dimension, CarerQol-7D VAS: Care-related Quality of Life-7 Dimensions Visual Analogue Scale, DS-DAT: Discomfort scale for patients with dementia of the Alzheimer type, ICECAP-O: ICEpop CAPability measure for Older people, VTE: Venous thromboembolism

^a^Adjusted for prespecified factors: baseline value of the investigated outcome, baseline NPI-NH score (binary, ≤12 vs >12) and long-term care organisation. ^b^A positive mean change corresponds with a deterioration from baseline; a negative mean change corresponds with an improvement from baseline. ^c^A positive mean change corresponds with an improvement from baseline; a negative mean change corresponds with a deterioration from baseline. ^d^An OR < 1.00 corresponds with a higher odd on faster cognitive decline in the usual care group; an OR > 1.00 corresponds with a higher odd on faster cognitive decline in the discontinuation group. ^e^An OR < 1.00 corresponds with a higher odd on a delirium in the usual care group; an OR > 1.00 corresponds with a higher odd on a delirium in the discontinuation group. ^f^An MR <1.00 corresponds with a higher ratio of falls in the usual care group; an MR >1.00 corresponds with a higher ratio of falls in the discontinuation group; ‡Hospital admission or emergency department visit with ischemic stroke, transient ischemic attack or venous thromboembolism; §Hospital admission or emergency department visit with intracranial, gastrointestinal (upper or lower) or other (primarily genitourinary and respiratory) bleeding; **#**These outcomes were retained as clinical-related because their measurement reflects changes in participants’ behavioural or clinical status necessitating the use of these particular medications.

# Supplementary Table 9. Detail of Medication related outcomes reported by Included Studies

| **Study identity** | **Recorded Time** | **Medication related outcome** | | | |
| --- | --- | --- | --- | --- | --- |
|  |  | **Change in Medication number** | **Changes in medication burden and complexity** | **Changes in inappropriate medication** | **Changes in adverse drug events** |
| Brunet *et al.^46^*, 2014 | At discharge | **Number of medications, mean**: BI: 7.27, AI: 4.82, p = **<0.05** |  |  |  |
| Hurley *et al.^6^*, 2024b | 6 months | **Total number of medications** (mean, SD): BI: 16 (6.1), AI: 15.4 (5.5), p = **.031** | **Drug burden Index** (median, IQR): BI: 1.03 (0.5-2.0), AI: 0.93 (0.5-1.8), p = **< .001** | **Modified medication appropriateness index**: (mean, SD): BI: 2.19 (0.73), AI: 2.08 (0.71), p = **<0.001** |  |
| Poudel *et al.^17^*, 2015 | NA | **Number of medications, mean**: BI: 9.6, AI: 9.3 |  |  |  |
| Frankenthal *et al. ^14^*, 2017 | 24 months | **Change in medication number, mean**: IG: 1.5, CG: -0.1, p = **0.03** |  | **Reduction in PIP, number of patients**: IG: 45, CG: 17, p =**0.02**  **Reduction in potential prescription omissions, number of patients**: IG: 6, CG: -8, p = 0.21 |  |
| Saad *et al. ^13^*, 2012 | At discharge | **Number of medications, mean (SD)**: BI: 7.7 (± 3.7), AI: 9.5 (± 3.6) |  |  |  |
| Kutner *et al.^8^,* 2015 |  | **Mean Number of Non-statin Medications**: IG: 10.1, CG: 10.8, p=**0.03** |  |  | **Statin-specific symptoms (muscle-related pain, weakness, headache, and fever) at six months** (in mean)**:** IG:7, C:7.2, p=0.71  **Adverse events:** 5% experienced non-serious adverse events |
| Bergh *et al.^12^*, 2012 | 25 weeks |  |  |  |  |
| Potter *et al.^15^*, 2016 | 12 months | **Change in regular medication number**, **mean±SD**: IG: -1.9 ± 4.1, CG: +0.1 ± 3.5, p=**0.04** |  |  |  |
| Suhrie *et al.^30^*, 2009 | At discharge |  |  | **Changes in un-necessary medication:** Admission: 1.7 ± 1.5 (74.2%) and at close-out: 0.6 ± 0.8 (39.3%), p = **<0.001** |  |
| M Chess-Williams *et al. ^10^*, 2024 | NR |  |  | **Change in PIM, per patients:** BI: 2.6 PIM, AI: 2.1 PIM |  |
| Gerardi *et al.^24^*, 2022 | 16 weeks |  |  | **Changes in inappropriate medication, n:** 45.8% of the inappropriate medicine was reduced after intervention (59 at baseline, 27 at 16 weeks) |  |
| Whitman *et al.^11^*, 2018 | After geriatric consultation | **Number of medications, mean**: BI: 12, AI: 9 |  | **Changes in PIM, mean:**  BI: 4.5, AI: 1.2 | **Symptoms or side effects at 14 mean days**: 18 out of 29 were available for follow up, where 16 patients previously reported symptoms or side effects reported reduction on it |
| Etherton-Beer *et al. ^16^*, 2023 | 12 months |  |  |  | **Frequency of Medication side effects, mean**±**SD:** IG-Open: 1.6 ± 3.4, IC: 1.9 ± 3.1, p = 0.45  IG-Blind: 1.5 ± 2.9, IC: 1.9 ± 3.1, p = 0.58  **Severity of Medication side effects, mean**±**SD:** IG-Open: 1.1 ± 2.7, IC: 1.2 ± 2.4, p = 0.88  IG-Blind: 0.9 ± 1.9, IC: 1.2 ± 2.4, p = 0.38 |
| Wauters *et al. ^28^,* 2021 | 1 month | **Change in medication:** Total medication decreased in 35.8% |  | **Changes in PIM**: At least one PIM reduced in 25.9% |  |
| Bogaerts *et al.^23^*, 2024 | 8 months |  |  |  | **Participants with reported SAE, n (%):** IG: 36(35.6%), CG: 25(24%), adjusted HR (95% CI) 1.65(0.98–2.79), p = 0.062 [Time to SAE in days, median (IQR): IG: 135(66-209), CG: 103(54-171)] |
| Whitty *et al.^34^*, 2018 | At discharge | **Change in medication number, mean + SD:** IC: 3.1**+**2.6, CC 0.9**+**1.5, p = <**0.001** |  |  |  |
| Ferro-Uriguen *et al.^47^*, 2023 | 6 months | **Change in regular medicine, mean (SD):** On T1 Patients: IG: -2.0(2.5), CG: -0.3(2.2), p = **0.013**  On T2 Patients: IG: -1.1(3.3), CG: 0.4(2.7), p = 0.196  **Change in no of patients with >10 medicines:** On T1 Patients: IG: -5, CG: -1, p = 0.147  On T2 Patients: IG: -1, CG: -4, p = 0.599 | **Change in DBI, mean (SD):** On T1 Patients: IG: -0.32(0.60), CG: -0.09(0.33), p = 0.088  On T2 Patients: IG: -0.11(0.72), CG: 0.21(0.51), p = 0.191  **Change in MRCI, mean (SD):** On T1 Patients: IG: -6.9(8.5), CG: 1.3(9.7), p = **0.002**  On T2 Patients: IG: -2.3(10.4), CG: 2.0(7.9), p = 0.239 | **Change in inappropriate medicine, mean (SD):** On T1 Patients: IG: -1.7(1.3), CG: -0.5(0.8), p = <**0.001**  On T2 Patients: IG: -1.3(1.3), CG: -0.4(0.7), p = 0.054 | **Change in DDIs, mean (SD):** On T1 Patients: IG: -1.3(1.7), CG: -0.4(2.8), p = 0.082  On T2 Patients: IG: -2.1(3.1), CG: 0.8(5.2), p = 0.116 |
| Choukroun *et al.^38^*, 2021 | NR | **Change in medication number, median (range):** BI: 10(5.5-12), AI: 8(5-11), p = 0.09 |  | **Number of inappropriate medications as per Laroche criteria (%):** BI: 16(31%), AI: 3(6%), p = **0.002**  **Number of inappropriate medications as per STOPP criteria (%):** BI: 29(57%), AI: 16(31%), p = 0.12  **Number of potential prescription omissions as per START criteria (%)**: BI: 34(67%), AI: 3(6%), p = **<0.001** | **Score on ADE geriatric risk score, median(range):** BI: 4(0-5), AI: 2(0-5), p = **0.02** |
| Kearney *et al.^44^*, 2023 | NR | **Change in medication number, median:** BI: 13.5, AI: 12, P = 0.28 |  |  |  |
| Suzuki *et al.^39^*, 2023 | Unclear | **Change in regular medicines (SD):** BI: 8.2(3.5), AI: 6.5 (1.4) |  |  | **Adverse drug events:** 44.7% (55/123) symptoms improved |
| Shirley *et al.^40^*, 2021 | NR | **Change in regular medicines:** BI: 15.5, AI: 12.6 |  |  |  |
| Pruskowski *et al.^41^*, 2017 | 4 months | **Change in medication number:** 10 medicines reduced after intervention |  |  |  |
| McIntyre *et al.^42^*, 2017 | 6 months | **Change in medication number, mean:** BI: 13.4, AI: 12.8 |  |  |  |

BI: Before Intervention, AI: After Intervention, SD: Standard deviation, IG: Intervention group, CG: Control or comparator group, IQR: Interquartile range, PIP: Potentially inappropriate prescribing, SAE: Serious Adverse events, T1: Dementia-like Trajectory Patients, T2: End stage organ failure trajectory, DDIs: Drug-drug interactions, DBI: Drug Burden Index, MRCI: Medication Regimen Complexity Index, ADE: Adverse drug events, HR: Hazardous Ratio,

# Supplementary Table 10. Detail of System outcomes reported by Included Studies

| **Study Identity (Author, year)** | **Recorded time (months)** | **System related outcomes** | | |
| --- | --- | --- | --- | --- |
|  |  | **Healthcare Expense** | **Satisfaction** | **Healthcare Utilisation** |
| Basri *et al ^1^*, 2018 | 4 months | **Cost saving with deprescribing**: $838.58 per intervention and $69,602.14 in total |  |  |
| Hurley *et al.^2^*, 2024a | 12 months | **Average cost saving with PIMs discontinuation**:  €619.56 (€61,336/99 patients)  **Nest cost benefit with deprescribing intervention**: €85,909  **Cost–benefit ratio**: 33.2 |  |  |
| Curtin *et al.*^3^, 2020 | 3 months | **Average cost per prescription, mean changed (SD):** IG: –$74.97 (148.32), CG: –$13.229 ($110.40), p = **.02** |  | **Emergency visit, proportion (95%CI)**: IG: 0.05(0.01,0.13), CG:0.08(0.03,0.17), Relative risk: 0.60(0.15,2.41), p=0.72  **Unplanned hospital admission, proportion (95%CI):** IG: 0.14(0.07,0.24), CG: 0.08(0.03,0.17), Relative Risk: 1.80(0.64,5.08). p=0.27  **Unscheduled medical reviews by GP, proportion (95%CI):** IG:0.61(0.47, 0.73), CG:0.57(0.43, 0.70), Relative Risk: 1.04(0.74, 1.45), p=0.82 |
| Kutner *et al. ^8^,* 2015 | Variable | **Average cost saving with statin discontinuation at 212.6 mean days**: $716.46 ($3.37 per day) | **Satisfaction with current health care at 6 months:** IG:4.63, CG:4.55, p=0.22 |  |
| Okafor *et al. ^29^*, 2024 | 12 months | **Cost saving with deprescribed, average per annum:** $7.32-$89.77, ($1.30 to $15.90 million if implemented in nationwide RACFs) |  |  |
| Frankenthal *et al.^14^*, 2017 | 24 months | **Medication cost difference, mean difference**: IG:113.3 (Israeli shekels), CG:10.2, p = <**0.001**) |  | **Hospitalisation, mean difference**: IG: -0.1, CG: 0.05, p = 0.5 |
| Garfinkel *et al.^25^*, 2007 | 12 months |  |  | **Referral rate to acute care facility, %:** IG: 11.8%, CG: 30%, p = **<0.002** |
| Whitman *et al.^11^*, 2018 | 14 mean days | **Healthcare expenditure reduction:** $111,390.00, or $4,282.27 per person |  |  |
| Nakagaito *et al. ^19^*, 2024 | 23.2 (Median months) | **Monthly medical cost for heart failure:** IG: 62,906 (502–187,246), CG: 29,236 (7920–180,305), p = 0.866. |  | **All-cause hospitalisation, n (%):** IG: 38(74.5%), CG: 93(57.8%), p=0.099  **All-cause hospitalisation events per year, median (IQR):** IG: 0.97 (0–1.50), CG: 0.50 (0–1.03), p = **0.030** |
| Gerardi *et al.^24^*, 2022 | 16 weeks |  | **Patient satisfaction:** 25/ 29 assessed, where only one patient expressed transient dissatisfaction due to return of symptom |  |
| Hurley *et al.^6^*, 2024b | 6 months | **Monthly medication cost in euro per patient** (mean, SD): BI: 186.8 (123.7), AI: 186.4 (121.2), p = .95 |  | **Emergency department visits per patients:** Mean difference (95% CI) = +0.03 (-0.08 to 0.02), p = 0.26  **Number of patients hospitalised**: BI: 8, AI: 6, 12.5% reduction |
| Tapper *et al. ^21^*, 2022 | 36 months |  |  | **Alcohol-Hospitalisation, %**: IG-Zolpidem: sHR (95% CI): 0.97 (0.80-1.18), p = 0.8  IG-Benzodiazepam: sHR (95% CI) = 0.98, p =0.8 |
| Potter *et al. ^15^*, 2016 | 12 months |  |  | **Hospital Admissions, proportion (95% CI)**: IG: 0.51 (0.37, 0.61), C: 0.50 (0.36, 0.63), p=0.99  **GP Attendance, proportion (95% CI)**: IG: 0.22 (0.12, 0.36), C: 0.10 (0.04, 0.23), p=0.16  **Call to GP, proportion (95% CI)**: IG: 0.53 (0.39, 0.67), C: 0.60 (0.46, 0.67), p=0.53 |
| Niznik *et al.^4^*, 2022 | 2 months |  |  | **Emergency department visit or hospitalisation, proportion (95%CI):** IG: 0.397 (0.351,0.449), CG: 0.398 (0.350,0.449), Risk difference: -0.001 (-0.070, 0.066), Risk ratio: 0.99(0.84,1.18) |
| Malik *et al. ^26^*, 2019 | 48 months |  |  | **HF readmission**: HR = 1.26, 95% CI: 1.08– 1.46, p = **0.003** (Higher risks in discontinuation group)  **All cause readmission:** HR = 1.15, 95% CI: 1.02–1.30, p = **0.026** (Higher risks in discontinuation group)  **Length of Hospital stay, days:** IG:5, CG: 5, p=0.332 |
| Etherton-Beer *et al. ^16^*, 2023 | 12 months |  |  | **Hospital admissions per resident, median (IQR)**: IG-Open: 0(1), IC: 0(1),  IG-Blind: 0(1), IC: 0(1) |
| Bogaerts *et al.^23^*, 2024 | 8 months |  |  | **Hospitalisation, n (%):** IG: 3(3%), CG: 1(1%) |
| Niznik *et al.^7^*, 2020b | 7.42 months |  |  | **All-cause-negative events** ^a^ **in 7.42 months** (events per person-year)**:** IG: 0.62, CG: 0.55, aOR=1.03,95% CI:0.97-1.10, p=0.34. |
| Whitty *et al. ^34^*, 2018 | 100 days | **Direct cost saving with medication stopping:** $1508.47, or $94.28 per 100 patient-days |  |  |
| Ferro-Uriguen *et al. ^47^*, 2023 | 1 month (28 day) | **Change in regular medicine cost, mean (SD)**: On T1 Patients: IG: -38.4(44.2), CG: 2.0(46.2), p = **0.004**  On T2 Patients: IG: -3.16(53.8), CG: 2.6(25), p = 0.760 |  |  |
| Kearney *et al.^44^*, 2023 | 12 months | **Cost saving with reduced hospitalisation:** (AUD$) 259,200 |  | **Unplanned admission-related bed days, median days (range):** IG: 1(0.0-3.25), CG: 2(1.75-4), p = **0.038**  **Hospitalisation, patient no (events)**: IG: 21(70), CG: 28(89) |
| Shirley *et al. ^40^*, 2021 | 14-30 days |  | **Patient Satisfaction**: 56% (14/25) responded satisfied |  |
| McIntyre *et al.^42^*, 2017 | 6 months |  | **Patient Satisfaction:** No patient reported any concerns (assessed based on symptom management) |  |

BI: Before Intervention, AI: After Intervention, SD: Standard deviation, IG: Intervention group, CG: Control or comparator group, IQR: Interquartile range, PIM: Potentially Inappropriate Medications, RR: Relative Risk, HR: Hazardous Ratio, sHR: subdistribution Hazardous Ration, aOR: adjusted Odd Ratio, HF: Heart Failure, IQR: Interquartile Range, CI: Confidence Interval, Qol: Quality of life,

# Critical Appraisal of Included Studies

## Supplementary Table 11. Quality assessment tool for Economic Evaluations

| **Included Studies** | **Criterion Number** | | | | | | | | | | | **Total Y (%)** |
| --- | --- | --- | --- | --- | --- | --- | --- | --- | --- | --- | --- | --- |
|  | **1** | **2** | **3** | **4** | **5** | **6** | **7** | **8** | **9** | **10** | **11** |  |
| Hurley *et al.^2^*, 2024a | Y | N | Y | Y | Y | Y | N | Y | Y | Y | Y | 9 (81) |

Y: Yes, N: Not, U: Unclear

Criterion No. 1. Is there a well-defined question? No. 2. Is there a comprehensive description of alternatives? No. 3. Are all important and relevant costs and outcomes for each alternative identified? No. 4. Has clinical effectiveness been established? No. 5. Are costs and outcomes measured accurately? No. 6. Are costs and outcomes valued credibly? No. 7. Are costs and outcomes adjusted for differential timing? No. 8. Is there an incremental analysis of costs and consequences? No. 9. Were sensitivity analyses conducted to investigate uncertainty in estimates of cost or consequences? No. 10. Do study results include all issues of concern to users? No. 11. Are the results generalizable to the setting of interest in the review?

## Supplementary Table 12. Quality assessment tool for Analytical Cross-Sectional Studies

| **Included Studies** | **Criterion Number** | | | | | | | | **Total Y (%)** |
| --- | --- | --- | --- | --- | --- | --- | --- | --- | --- |
|  | **1** | **2** | **3** | **4** | **5** | **6** | **7** | **8** |  |
| M Chess-Williams *et al. ^10^*, 2024 | Y | Y | Y | Y | N | N | Y | Y | 6 (75) |

Y: Yes, N: Not, U: Unclear

Criterion No. 1. Were the criteria for inclusion in the sample clearly defined? No. 2. Were the study subjects and the setting described in detail? No. 3. Was the exposure measured in a valid and reliable way? No. 4. Were objective, standard criteria used for measurement of the condition? No. 5. Were confounding factors identified? No. 6. Were strategies to deal with confounding factors stated? No. 7. Were the outcomes measured in a valid and reliable way? No. 8. Was appropriate statistical analysis used?

## Supplementary Table 13. Quality assessment tool for Randomised control study

| **Included Studies** | **Criterion Number** | | | | | | | | | | | | | **Total Y (%)** |
| --- | --- | --- | --- | --- | --- | --- | --- | --- | --- | --- | --- | --- | --- | --- |
|  | **1** | **2** | **3** | **4** | **5** | **6** | **7** | **8** | **9** | **10** | **11** | **12** | **13** |  |
| Curtin *et al.*^3^, 2020 | Y | Y | Y | N | N | Y | Y | Y | Y | Y | Y | Y | Y | 11 (85) |
| Kutner *et al. ^8^*, 2015 | Y | Y | N | N | N | Y | Y | Y | Y | Y | Y | Y | Y | 10 (77) |
| Tse *et al. ^9^*, 2008 | Y | N | N | Y | Y | Y | Y | Y | Y | Y | Y | Y | Y | 11 (85) |
| Etherton-Beer *et al. ^16^*, 2023 | Y | Y | Y | Y | Y | Y | Y | Y | Y | Y | Y | Y | Y | 13 (100) |
| Okafor *et al. ^29^*, 2024 | Y | Y | Y | Y | Y | Y | Y | Y | Y | Y | Y | Y | Y | 13 (100) |
| Potter *et al. ^15^*, 2016 | Y | Y | Y | N | N | Y | N | Y | Y | Y | Y | Y | Y | 10 (77) |
| Ruths *et al. ^22^*, 2004 | Y | Y | Y | Y | Y | Y | Y | Y | Y | Y | Y | Y | Y | 13 (100) |
| Bogaerts *et al. ^23^*, 2024 | Y | Y | Y | N | N | Y | Y | Y | Y | Y | Y | Y | Y | 11 (85) |
| Frankenthel *et al.^14,43^*, 2017 | Y | Y | Y | N | N | Y | Y | Y | Y | Y | Y | Y | Y | 11 (85) |
| Bergh *et al. ^12^*, 2012 | Y | Y | Y | Y | Y | Y | Y | Y | Y | Y | Y | Y | Y | 13 (100) |
| Ferro-Uriguen *et al. ^47^*, 2023 | Y | N | U | N | N | Y | U | Y | Y | Y | Y | Y | Y | 8 (62) |

Y: Yes, N: Not, U: Unclear, N/A: Not Available

Criterion No. 1: Was true randomization used for assignment of participants to treatment groups? No. 2: Was allocation to groups concealed? No. 3: Were treatment groups similar at the baseline? No. 4: Were participants blind to treatment assignment? No. 5: Were those delivering the treatment blind to treatment assignment? No. 6: Were treatment groups treated identically other than the intervention of interest? No. 7: Were outcome assessors blind to treatment assignment? No. 8: Were outcomes measured in the same way for treatment groups? No 9: Were outcomes measured in a reliable way? No. 10: Was follow up complete and if not, were differences between groups in terms of their follow up adequately described and analysed? No. 11: Were participants analysed in the groups to which they were randomized? No. 12: Was appropriate statistical analysis used? No. 13: Was the trial design appropriate and any deviations from the standard RCT design (individual randomization, parallel groups) accounted for in the conduct and analysis of the trial?

## Supplementary Table 14. Quality assessment of Cohort Studies

| **Included Studies** | **Criterion Number** | | | | | | | | | | | **Total Y (%)** |
| --- | --- | --- | --- | --- | --- | --- | --- | --- | --- | --- | --- | --- |
|  | **1** | **2** | **3** | **4** | **5** | **6** | **7** | **8** | **9** | **10** | **11** |  |
| Niznik *et al.^4^*, 2022 | Y | Y | Y | Y | Y | Y | Y | Y | Y | Y | Y | 11 (100) |
| Niznik *et al.^5^*, 2020a | N | Y | Y | Y | Y | Y | Y | Y | Y | Y | Y | 10 (91) |
| Niznik *et al.^7^*, 2020b | N | Y | Y | Y | Y | Y | Y | Y | Y | Y | Y | 10 (91) |
| Tapper *et al. ^21^*, 2022 | Y | Y | Y | Y | Y | Y | Y | Y | N | Y | Y | 10 (91) |
| Malik *et al. ^26^*, 2019 | Y | Y | U | Y | Y | Y | Y | Y | Y | Y | Y | 10 (91) |
| Daiello *et al. ^27^*, 2009 | Y | Y | Y | N | U | Y | Y | Y | N | U | Y | 7 (64) |
| Brunetti *et al. ^18^*, 2024 | N | Y | Y | Y | Y | Y | Y | Y | Y | Y | Y | 10 (91) |
| Nakagaito *et al. ^19^*, 2024 | N | Y | Y | Y | Y | Y | Y | Y | U | Y | Y | 9 (82) |
| Yeh *et al. ^31^*, 2013 | Y | Y | Y | N | N | Y | Y | Y | N | Y | Y | 8 (73) |
| Czikk *et al. ^33^*, 2022 | NA | NA | Y | U | N | Y | Y | Y | Y | Y | Y | 7 (64) |
| Hayes *et al. ^32^*, 2023 | Y | Y | Y | Y | Y | Y | Y | Y | U | Y | Y | 10 (91) |
| Riveras *et al. ^35^*, 2024 | U | Y | Y | N | N | Y | Y | Y | Y | Y | Y | 8 (73) |
| Chin-Yee *et al. ^36^*, 2022 | N | Y | Y | Y | Y | Y | Y | Y | N | N | Y | 8 (73) |
| Ruderman *et al. ^45^*, 2018 | Y | Y | Y | Y | Y | Y | Y | Y | Y | Y | Y | 11 (100) |
| Caravaca *et al. ^20^*, 2018 | NA | NA | Y | Y | Y | Y | Y | Y | Y | Y | Y | 9 (82) |

Y: Yes, N: Not, U: Unclear, NA: Not available

Criterion No. 1. Were the two groups similar and recruited from the same population? No. 2. Were the exposures measured similarly to assign people to both exposed and unexposed groups? No. 3. Was the exposure measured in a valid and reliable way? No. 4. Were confounding factors identified? No. 5. Were strategies to deal with confounding factors stated? No. 6. Were the groups/participants free of the outcome at the start of the study (or at the moment of exposure)? No. 7. Were the outcomes measured in a valid and reliable way? No. 8. Was the follow-up time reported and sufficient to be long enough for outcomes to occur? No. 9. Was follow-up complete, and if not, were the reasons for loss to follow-up described and explored? No. 10. Were strategies to address incomplete follow-up utilized? No. 11 Was appropriate statistical analysis used?

## Supplementary Table 15. Quality assessment tool for Quasi-Experimental Studies

| **Included Studies** (Author, year) | **Criterion Number** | | | | | | | | | **Total Y (%)** |
| --- | --- | --- | --- | --- | --- | --- | --- | --- | --- | --- |
|  | **1** | **2** | **3** | **4** | **5** | **6** | **7** | **8** | **9** |  |
| Basri *et al. ^1^*, 2018 | Y | NA | NA | NA | NA | NA | Y | Y | Y | 4 (44) |
| Brunet *et al.^46^*, 2014 | Y | NA | NA | NA | Y | Y | Y | Y | Y | 6 (67) |
| Hurley *et al.^6^*, 2024b | Y | NA | NA | NA | Y | Y | Y | Y | Y | 6 (67) |
| Poudel *et al. ^17^*, 2015 | Y | NA | NA | NA | Y | Y | Y | Y | Y | 6 (67) |
| Wauters *et al. ^28^*, 2021 | Y | NA | NA | NA | Y | Y | Y | Y | Y | 6 (67) |
| Gerardi *et al. ^24^*, 2022 | Y | NA | NA | NA | Y | Y | Y | Y | Y | 6 (67) |
| Garfinkel *et al. ^25^*, 2007 | Y | Y | Y | Y | N | Y | Y | U | Y | 7 (78) |
| Whitman *et al. ^11^*, 2018 | Y | NA | NA | NA | Y | Y | Y | Y | Y | 6 (67) |
| Saad *et al. ^13^*, 2012 | Y | NA | NA | NA | Y | Y | Y | Y | Y | 6 (67) |
| Suhrie *et al. ^30^*, 2009 | Y | NA | NA | NA | Y | Y | Y | Y | Y | 6 (67) |
| Whitty *et al. ^34^*, 2018 | Y | Y | N | Y | Y | Y | Y | Y | Y | 7 (78) |
| Bravo-Jose *et al. ^37^*, 2019 | Y | NA | NA | NA | Y | Y | Y | Y | Y | 6 (67) |
| Choukroun *et al. ^38^*, 2021 | Y | NA | NA | NA | Y | Y | Y | U | Y | 5 (56) |
| Kearney *et al. ^44^*, 2023 | Y | Y | N | Y | Y | Y | Y | Y | Y | 8 (89) |
| Suzuki *et al. ^39^*, 2023 | Y | NA | NA | NA | Y | Y | Y | N | Y | 5 (56) |
| Shirley *et al. ^40^*, 2021 | Y | NA | NA | NA | Y | Y | Y | Y | Y | 6 (67) |
| Pruskowski *et al. ^41^*, 2017 | Y | NA | NA | NA | Y | Y | Y | Y | Y | 6 (67) |
| McIntyre *et al. ^42^*, 2017 | Y | NA | NA | NA | Y | Y | Y | Y | Y | 6 (67) |

Y: Yes, N: Not, U: Unclear, NA: Not applicable due to study design

Criterion No. 1: Is it clear in the study what is the “cause” and what is the “effect” (i.e. there is no confusion about which variable comes first)? No. 2: Was there a control group? No. 3: Were participants included in any comparisons similar? No. 4: Were the participants included in any comparisons receiving similar treatment/care, other than the exposure or intervention of interest? No. 5: Were there multiple measurements of the outcome, both pre and post the intervention/exposure? No. 6: Were the outcomes of participants included in any comparisons measured in the same way? No. 7: Were outcomes measured in a reliable way? No. 8: Was follow-up complete and if not, were differences between groups in terms of their follow-up adequately described and analysed? No 9: Was appropriate statistical analysis used?

**References**

1. Basri DS, DiScala SL, Brooks AT, et al. Analysis of Inpatient Hospice Pharmacist Interventions Within a Veterans Affairs Medical Center. *J Pain Palliat Care Pharmacother* 2018; 32: 240–247.

2. Hurley E, Byrne S, Walsh E, et al. Cost avoidance of pharmacist-led deprescribing using STOPPFrail for older adults in nursing homes (a). *Int J Clin Pharm* 2024; 46: 1163–1171.

3. Curtin D, Jennings E, Daunt R, et al. Deprescribing in Older People Approaching End of Life: A Randomized Controlled Trial Using STOPPFrail Criteria. *J Am Geriatr Soc* 2020; 68: 762–769.

4. Niznik JD, Zhao X, Slieanu F, et al. Effect of Deintensifying Diabetes Medications on Negative Events in Older Veteran Nursing Home Residents. *Diabetes Care* 2022; 45: 1558–1567.

5. Niznik JD, Zhao X, He M, et al. Impact of deprescribing AChEIs on aggressive behaviors and antipsychotic prescribing (a). *Alzheimers Dement* 2020; 16: 630–640.

6. Hurley E, Dalton K, Byrne S, et al. Pharmacist-Led Deprescribing Using STOPPFrail for Frail Older Adults in Nursing Homes (b). *J Am Med Dir Assoc*; 25. Epub ahead of print 1 September 2024. DOI: 10.1016/j.jamda.2024.105122.

7. Niznik JD, Zhao X, He M, et al. Risk for Health Events After Deprescribing Acetylcholinesterase Inhibitors in Nursing Home Residents With Severe Dementia (b). *J Am Geriatr Soc* 2020; 68: 699–707.

8. Kutner JS, Blatchford PJ, Taylor DH, et al. Safety and benefit of discontinuing statin therapy in the setting of advanced, life-limiting illness: a randomized clinical trial. *JAMA Intern Med* 2015; 175: 691–700.

9. Tse W, Frisina PG, Hälbig TD, et al. The effects of withdrawal of dopaminergic medication in nursing home patients with advanced parkinsonism. *J Am Med Dir Assoc* 2008; 9: 670–5.

10. M Chess-Williams L, M Broadbent A, Hattingh L. Cross-sectional study to evaluate patients’ medication management with a new model of care: incorporating a pharmacist into a community specialist palliative care telehealth service. *BMC Palliat Care* 2024; 23: 172.

11. Whitman A, DeGregory K, Morris A, et al. Pharmacist-led medication assessment and deprescribing intervention for older adults with cancer and polypharmacy: a pilot study. *Support Care Cancer* 2018; 26: 4105–4113.

12. Bergh S, Selbæk G, Engedal K. Discontinuation of antidepressants in people with dementia and neuropsychiatric symptoms (DESEP study): double blind, randomised, parallel group, placebo controlled trial. *BMJ* 2012; 344: e1566.

13. Saad M, Harisingani R, Katinas L. Impact of geriatric consultation on the number of medications in hospitalized older patients. *Consult Pharm* 2012; 27: 42–8.

14. Frankenthal D, Israeli A, Caraco Y, et al. Long-Term Outcomes of Medication Intervention Using the Screening Tool of Older Persons Potentially Inappropriate Prescriptions Screening Tool to Alert Doctors to Right Treatment Criteria. *J Am Geriatr Soc* 2017; 65: e33–e38.

15. Potter K, Flicker L, Page A, et al. Deprescribing in Frail Older People: A Randomised Controlled Trial. *PLoS One* 2016; 11: e0149984.

16. Etherton-Beer C, Page A, Naganathan V, et al. Deprescribing to optimise health outcomes for frail older people: a double-blind placebo-controlled randomised controlled trial—outcomes of the Opti-med study. *Age Ageing* 2023; 52: 1–10.

17. Poudel A, Peel NM, Mitchell CA, et al. Geriatrician interventions on medication prescribing for frail older people in residential aged care facilities. *Clin Interv Aging* 2015; 10: 1043–1051.

18. Brunetti E, Presta R, Okoye C, et al. Predictors and Outcomes of Oral Anticoagulant Deprescribing in Geriatric Inpatients With Atrial Fibrillation: A Retrospective Multicenter Cohort Study. *J Am Med Dir Assoc* 2024; 25: 545-551.e4.

19. Nakagaito M, Imamura T, Ushijima R, et al. The Impact of the Withdrawal of SGLT2 Inhibitors on Clinical Outcomes in Patients with Heart Failure. *J Clin Med* 2024; 13: 3196.

20. Caravaca F, Caravaca-Fontán F, Azevedo L, et al. Changes in renal function after discontinuation of vitamin D analogues in advanced chronic kidney disease. *Nefrología* 2018; 38: 179–189.

21. Tapper EB, Zhao Z, Winder GS, et al. Deprescribing zolpidem reduces falls and fractures in patients with cirrhosis. *JHEP Reports* 2022; 4: 100478.

22. Ruths S, Straand J, Nygaard HA, et al. Effect of antipsychotic withdrawal on behavior and sleep/wake activity in nursing home residents with dementia: a randomized, placebo-controlled, double-blinded study. The Bergen District Nursing Home Study. *J Am Geriatr Soc* 2004; 52: 1737–43.

23. Bogaerts JMK, Gussekloo J, de Jong-Schmit BEM, et al. Effects of the discontinuation of antihypertensive treatment on neuropsychiatric symptoms and quality of life in nursing home residents with dementia (DANTON): a multicentre, open-label, blinded-outcome, randomised controlled trial. *Age Ageing*; 53. Epub ahead of print 2 July 2024. DOI: 10.1093/ageing/afae133.

24. Gerardi S, Sperlea D, Levy SO-L, et al. Implementation of targeted deprescribing of potentially inappropriate medications in patients on hemodialysis. *Am J Health Syst Pharm* 2022; 79: S128–S135.

25. Garfinkel D, Zur-Gil S, Ben-Israel J. The war against polypharmacy: a new cost-effective geriatric-palliative approach for improving drug therapy in disabled elderly people. *Isr Med Assoc J* 2007; 9: 430–4.

26. Malik A, Masson R, Singh S, et al. Digoxin Discontinuation and Outcomes in Patients With Heart Failure With Reduced Ejection Fraction. *J Am Coll Cardiol* 2019; 74: 617–627.

27. Daiello LA, Ott BR, Lapane KL, et al. Effect of discontinuing cholinesterase inhibitor therapy on behavioral and mood symptoms in nursing home patients with dementia. *Am J Geriatr Pharmacother* 2009; 7: 74–83.

28. Wauters M, Elseviers M, Vander Stichele R, et al. Efficacy, feasibility and acceptability of the OptiMEDs tool for multidisciplinary medication review in nursing homes. *Arch Gerontol Geriatr* 2021; 95: 104391.

29. Okafor CE, Keramat SA, Comans T, et al. Cost-Consequence Analysis of Deprescribing to Optimize Health Outcomes for Frail Older People: A Within-Trial Analysis. *J Am Med Dir Assoc* 2024; 25: 539-544.e2.

30. Suhrie EM, Hanlon JT, Jaffe EJ, et al. Impact of a geriatric nursing home palliative care service on unnecessary medication prescribing. *Am J Geriatr Pharmacother* 2009; 7: 20–5.

31. Yeh Y-C, Liu C-L, Peng L-N, et al. Potential benefits of reducing medication-related anticholinergic burden for demented older adults: a prospective cohort study. *Geriatr Gerontol Int* 2013; 13: 694–700.

32. Hayes KN, Zhang T, Kim DH, et al. Benefits and Harms of Standard Versus Reduced‐Dose Direct Oral Anticoagulant Therapy for Older Adults With Multiple Morbidities and Atrial Fibrillation. *J Am Heart Assoc* 2023; 12: 29865.

33. Czikk D, Parpia Y, Roberts K, et al. De-Prescribing Proton Pump Inhibitors in Patients With End Stage Kidney Disease: A Quality Improvement Project. *Can J Kidney Health Dis*; 9. Epub ahead of print 26 January 2022. DOI: 10.1177/20543581221106244.

34. Whitty R, Porter S, Battu K, et al. A pilot study of a Medication Rationalization (MERA) intervention. *CMAJ Open* 2018; 6: E87–E94.

35. Riveras A, Crul M, van der Kloes J, et al. A Tool for Deprescribing Antithrombotic Medication in Palliative Cancer Patients: A Retrospective Evaluation. *J Pain Palliat Care Pharmacother* 2024; 38: 20–27.

36. Chin-Yee N, Gomes T, Tanuseputro P, et al. Anticoagulant use and associated outcomes in older patients receiving home palliative care: a retrospective cohort study. *CMAJ* 2022; 194: E1198–E1208.

37. Bravo-José P, Sáez-Lleó CI, Peris-Martí JF. Deprescribing antipsychotics in long term care patients with dementia. *Farm Hosp* 2019; 43: 140–145.

38. Choukroun C, Leguelinel-Blache G, Roux-Marson C, et al. Impact of a pharmacist and geriatrician medication review on drug-related problems in older outpatients with cancer. *J Geriatr Oncol* 2021; 12: 57–63.

39. Uchida M, Suzuki S, Sugawara H, et al. Multicentre prospective observational study on community pharmacist interventions to reduce inappropriate medications. *Int J Pharm Pract* 2023; 30: 427–433.

40. Shirley L, DiScala S, Brooks A, et al. Pilot of a pharmacist-integrated interprofessional team to optimize prescribing in a telemedicine palliative care clinic. *JACCP:  JOURNAL OF THE AMERICAN COLLEGE OF CLINICAL PHARMACY* 2021; 4: 1093–1099.

41. Pruskowski J, Handler SM. The DE-PHARM Project: A Pharmacist-Driven Deprescribing Initiative in a Nursing Facility. *Consult Pharm* 2017; 32: 468–478.

42. McIntyre C, McQuillan R, Bell C, et al. Targeted Deprescribing in an Outpatient Hemodialysis Unit: A Quality Improvement Study to Decrease Polypharmacy. *Am J Kidney Dis* 2017; 70: 611–618.

43. Frankenthal D, Lerman Y, Kalendaryev E, et al. Intervention with the screening tool of older persons potentially inappropriate prescriptions/screening tool to alert doctors to right treatment criteria in elderly residents of a chronic geriatric facility: a randomized clinical trial. *J Am Geriatr Soc* 2014; 62: 1658–65.

44. Kearney A, Tiwari N, Cullen O, et al. Improving palliative and supportive care in advanced cirrhosis: the HepatoCare model of integrated collaborative care. *Intern Med J* 2023; 53: 1963–1971.

45. Ruderman I, Smith ER, Toussaint ND, et al. Longitudinal changes in bone and mineral metabolism after cessation of cinacalcet in dialysis patients with secondary hyperparathyroidism. *BMC Nephrol* 2018; 19: 113.

46. Molist Brunet N, Sevilla-Sánchez D, Amblàs Novellas J, et al. Optimizing drug therapy in patients with advanced dementia: A patient-centered approach. *Eur Geriatr Med* 2014; 5: 66–71.

47. Ferro-Uriguen A, Beobide-Telleria I, Gil-Goikouria J, et al. Effectiveness of a Person-Centered Prescription Model in Hospitalized Older People at the End of Life According to Their Disease Trajectories and Frailty Index. *Int J Environ Res Public Health* 2023; 20: 3542.
